# Highly conductive and durable nanocomposite hard coatings of carbon fiber reinforced thermoplastic composites against lightning strikes

**DOI:** 10.1186/s11671-024-04041-5

**Published:** 2024-06-06

**Authors:** Clay Parten, Balakrishnan Subeshan, Ramazan Asmatulu

**Affiliations:** Department of Mechanical Engineering, Wichita State University, 1845 Fairmount, Wichita, KS 67260 USA

**Keywords:** Conductive coatings, Lightning strike protection, Thermoplastic composites, Aircraft structures

## Abstract

The growing use of thermoplastic composites (TPCs) like low-melting polyaryletherketone (LM-PAEK) matrices reinforced with unidirectional carbon fiber (CF) in aircraft structures presents a significant challenge in terms of lightning strikes and electromagnetic interference shielding during aircraft operations. This is due to the weak electrical conductivity of TPC structures, which results in widespread damage when struck by lightning. The repair and maintenance of these extended damaged sites can increase operational costs and loss of flights. Several lightning strike protection (LSP) systems have been developed and implemented to address these concerns. This study evaluated a highly conductive coating with a low filler rate for its effectiveness as an LSP solution for TPCs on exterior aircraft surfaces. The TPC panel without any coatings was first studied. Subsequently, the level of conductivity was increased by incorporating the nanoscale conductive fillers, silver-coated copper (Ag/Cu) nanoflakes, with a silver content of 20 wt.% (Ag20/Cu) and 30 wt.% (Ag30/Cu), correspondingly, into the coating at two loadings of 55 wt.% and 70 wt.% in an epoxy carrier for the surface coatings. The behavior of electrical and surface conductivity was thoroughly examined to understand the impact of Ag/Cu with a high aspect ratio and the effectiveness of the LSP solution. In addition, the spray-coated TPC panels underwent rigorous Zone 2A lightning strike testing using simulated lightning current, in agreement with the industry standard of Society of Automotive Engineers (SAE) Aerospace Recommended Practice (ARP) 5412B. Despite the higher resistance due to the lower conductive coating weight, the TPC panels with Ag30/Cu at loading of 70 wt.% achieved better results than those with Ag30/Cu at loading of 55 wt.%. This is evidenced by the minor structural delamination and CF breakage on the front surface, which proposes a new economic route for a sustainable post-processed LSP system in the aviation industry.

## Introduction

Thermoplastic composites (TPCs) have been used extensively in the aerospace industry for aircraft structural parts in recent years due to their lightweight nature, high strength, ability to adjust mechanical properties, and resistance to corrosion [1–4]. In the past, traditional metal frames on aircraft were highly successful in dissipating lightning strike currents and protecting against their direct and indirect impacts, owing to their high electrical conductivity. However, TPCs are gradually replacing these conventional metal frames in modern aircraft design [5, 6]. The low and anisotropic electrical and thermal conductivity of TPC laminates has long been a challenge in their use in the aerospace industry [7, 8]. This can be especially problematic in the event of a lightning strike, where TPCs exhibit much lower electrical conductivity (ranging from 10^2^ to 10^4^ S/m in the in-plane direction and 10^–4^ S/m in the in-thickness direction) and thermal conductivity (ranging from ~ 10^–1^ W/m℃) compared to metals like aluminum, steel, and titanium alloy, which boast much higher electrical conductivity (10^6^–10^8^ S/m) and thermal conductivity (10^2^ W/m℃) [9–12]. The challenge of effectively transferring and dissipating the substantial amounts of charge and heat associated with a lightning strike is the trouble of the low electrical conductivity of TPCs. The poor electrical conductivity of TPC laminates, in comparison to metallic materials, exacerbates the problem, leading to an increase in local temperature which can result in severe damage, such as fiber sublimation, resin pyrolysis, resistive heating, deep delamination, less variation with all intralaminar and interlaminar properties and overpressure, which can exaggerate structural degradation and disintegration [13–20].

Lightning strikes happen most frequently during critical phases of aircraft, such as takeoff and landing, or when an aircraft moves through storm clouds. During a lightning strike, the current seeks to travel through the most conductive path within the TPC structure, typically the shortest path between two extremal points on the aircraft [21]. When a high electrical discharge occurs, the air that is usually almost insulating becomes ionized and transforms into plasma, which has a highly effective conducting ability. This leads to the formation of a lightning channel, where the temperature rapidly increases due to the intense energy transfer. The process of plasma formation is further intensified by the heat generated as a result of the TPC structure acting as a low-conducting material [22]. When a massive electrical discharge occurs, some energy is released in the form of heat, which can reach incredibly high temperatures of up to several thousand Celsius in the case of plasma channels. In this context, it is important that the temperature within the arc root, delineating the space charge zone situated between the electrode and the arc column, remains constrained below the threshold of the material's sublimation or evaporation temperature. As the heat energy progressively accumulates, a lightning plasma leader swiftly emerges from the cathode tip, traversing towards the anode plate, which is accompanied by a notable escalation in the electrical conductivity of the surrounding air. Subsequently, thermal plasma, characterized by its intense heat and ionized composition, impinges upon and stagnates on the surface of the anode plate. This interaction prompts the expansion of the arc root after its initial attachment. Concurrently, an overpressure manifests within the attachment region.

**Fig. 1:**
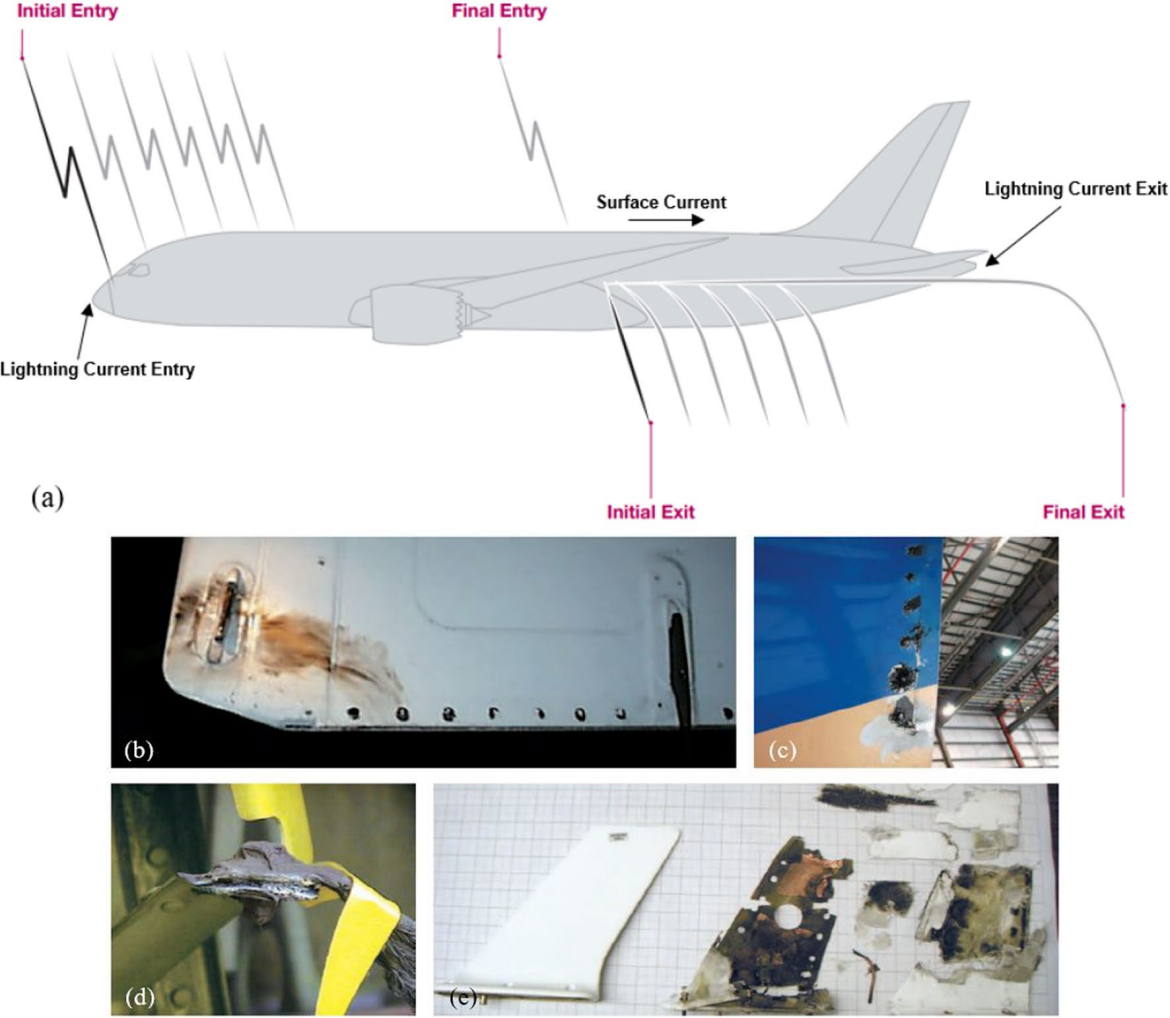
Images showing **a** the possible lightning strike on the aircraft, and various incurred damages on **b** horizontal stabilizer, **c** rudder, **d** antenna, and **e** bond jumper [26]

This intense heat flux can cause the polymeric matrix of the material to vaporize, as well as ablate the reinforcement fibers and even ignite the structure itself. This means that the heat generated by a lightning strike can cause significant damage to the structural integrity of an aircraft, potentially leading to structural failure or other safety hazards [23, 24]. Among the effects resulting from the interaction between lightning and aircraft, the "swept effect" emerges as a particularly destructive occurrence, which materializes when the coupling point of a lightning strike undergoes a positional shift owing to the aircraft's relative motion during flight, as depicted in Fig. 1, which depicts the process by which lightning can be originated at the leading edges of an aircraft due to ionization, which creates an opportunity for a lightning strike [25]*.* As mentioned before, lightning currents then travel along the length of the aircraft and exit through the tail, forming a circuit with the aircraft. As demonstrated in Fig. 1b–e, significant damages can occur if the aircraft is struck by lightning [26].

The aerospace industry has explored various LSP system solutions for preserving the TPC structures from lightning strikes. To mitigate the harm caused by lightning strikes on aircraft structures made of TPCs, various lightning strike protection (LSP) systems have been developed, including metallic surface inserts. These systems aim to reduce damage and are implemented through the application of metallic foil, perforated metallic foil, or expanded metal foil on the surface of the TPC structure [27–30]. A comprehensive review by Gagne et al. (2014) analyzed the variety of LSP systems that could be utilized, while Karch et al. (2015) conducted a review of the conventional metal-based LSPs, the lightning current waveform, the probability of lightning strike based on the zoning of aircraft, and the advancement in composite LSP systems [31–33]. However, despite the benefits that LSP systems offer for protecting TPC structures from lightning strikes, their implementation also comes with some drawbacks. The addition of metallic inserts to the TPC surface, for example, increases the overall weight of the structure, making it less lightweight and potentially compromising its structural integrity [34–38]. Furthermore, incorporating these systems into the composite manufacturing process can also be complex and time-consuming. Therefore, improving the electrical conductivity of TPCs remains a demanding task that researchers and scientists in the aerospace industry are continuously striving to address.

Moreover, Wang et al. [39] used reduced graphene oxides (RGO) to improve the conductivity of the TPC structure and provide LSP protection by adding them to the surface. Similarly, Zhang et al. [40] used a galvanic corrosion method to enhance the conductivity of the LSP material in the composite by incorporating graphene, carbon fiber (CF), and indium tin oxide (ITO) into aluminum and copper (Al/Cu) meshes. In addition, a non-woven film of carbon nanotubes (CNTs), known as a carbon nanotube film (CNF), has also been proposed as an alternative solution for protecting TPC structures from lightning strikes. The low density of CNF makes it a more attractive alternative, with its weight much lower at just 0.83 g/cm^3^ compared to other metals (Al (2.7 g/cm^3^) and Cu (8.9 g/cm^3^)) [41–43]. Furthermore, the interface compatibility of CNF with various resins during the TPC manufacturing process is better than metallic mesh. Hence, CNF, as a promising alternative to metallic mesh, can be applied in LSP applications [44–46].

The electrical conductivity improvement of TPC can be achieved by integrating conductive fillers. One solution is to utilize inherently conductive polymers (ICPs) as conductive fillers in the TPC structure [47]. The low electrical conductivity of TPC is primarily attributed to the dielectric presence of the polymer matrix. The introduction of conductive fillers can offer a promising alternative to lower the damage caused by lightning current and overcome the disadvantages associated with traditional LSP systems, such as added weight and increased manufacturing complexity [48, 49]. Many studies have been carried out with a focus on the dispersion of carbon fillers, including carbon black, carbon nanotubes (CNTs), and carbon nanofibers [50–54]. The electrical conductivity is improved by incorporating these conductive fillers, which show conductive performance above a certain percolation threshold that depends on the aspect ratio of the particles. Balberg et al. and Kirkpatrick et al. described this percolation threshold [55, 56]. In order to reduce the damage from lightning strikes on TPC structures, a significant amount of research has been dedicated to enhancing the electrical conductivity of the TPC laminates through a variety of LSP measures, which include improving both the surface and in-plane electrical conductivity, as well as the electrical conductivity throughout the thickness of the TPC [57–59].

The integration of conductive nanofillers into the matrix of CF has garnered attention as a promising strategy to increase electrical conductivity without imposing notable weight increments. [60]. For example, Senis et al. demonstrated this concept by incorporating graphene oxide (GO) at a volume fraction of 6.3% into an epoxy resin matrix. This deliberate inclusion resulted in a remarkable 200% enhancement in the through-thickness electrical conductivity [61]. Raj et al. conducted a study involving the in-situ alignment of conductive nanofillers, such as CFs, within epoxy composites under the influence of an alternating current (AC) electrical field. Through this procedure, the electrical conductivity of epoxy composites exhibited a substantial enhancement by approximately 7 to 8 orders of magnitude [62]. However, it is notable to mention that as the concentration of conductive nanofillers increased within the composite, the relative improvement in electrical conductivity became less prominent. Of significant concern is the potential impact of elevated loadings of conductive nanomaterials within the matrix, which can result in nonuniform distribution throughout the composite and contribute to the formation of a highly viscous, uncured epoxy mixture. Such a viscous mixture poses considerable challenges during the infusion process into carbon fabrics, complicating manufacturing procedures.

During a lightning strike, aircraft can be subjected to a current as high as 200 kA or a voltage exceeding millions of volts, which have the potential to vaporize metals and cause punctures, burn-throughs, and other types of damage to the composite materials. The lightning damage on TPC laminates can be an intricate physical activity involving numerous physical fields, for instance, the high-voltage shockwave, Joule heat, and electromagnetic force generated by the strong current [63, 64]. Therefore, applying the lightning current components as a sequence contained by the given test shot instead of distinct shots is essential to simulate these complex conditions accurately. As indicated by the lightning test methods mentioned in the Society of Automotive Engineers (SAE) Aerospace Recommended Practice (ARP) 5412B and ARP5416, the TPCs used in aircraft should undergo a cyclic and sequential lightning test with multiple waveforms [65, 66]. This test should comprise four components: the initial stroke, intermediate current, continuing stroke, and subsequent return stroke. Despite the recommended lightning test method outlined in the standards, most studies performed on TPC lightning protection have utilized a single lightning current component or a current impulse different from the waveforms specified in the SAE ARP5412B [66]. The materials and methods section explained more about the lightning strike tests.

The objective of this work was to evaluate the LSP performance of the TPC panels fabricated with unidirectional CF. This study aimed to find the optimal combination of processing methods and nanomaterials to enhance the resistivity of LSP systems while maintaining the surface durability of the epoxy matrix with minimal weight penalties and maximizing the electrical conductivity for perfect filler content. This was achieved by focusing on two variations of silver-coated copper (Ag/Cu) nanoflakes, Ag20/Cu and Ag30/Cu, into the coating at two loadings of 55 wt.% and 70 wt.% in an epoxy carrier [67]. The selection of the conductive nanofiller in the coating formulation was driven to enhance the effectiveness of LSP systems on TPC aircraft structures. While CNF has been extensively studied and offers desirable properties, including high conductivity, alternative materials were explored to address potential cost and performance considerations. The decision to investigate a new coating formulation incorporating metallic nanoflakes of Ag and Cu was capitalized due to several factors. Firstly, Ag and Cu are renowned for their excellent electrical and thermal conductivities and combining them in the coating matrix was hypothesized to yield a synergistic effect, potentially enhancing the overall conductivity compared to coatings containing only one type of metal. It is also expected that during lightning strikes, the current should be on the top surface of the fiber composites, so adding electrically and thermally conductive nanocomposite top surface coatings will satisfy this expectation. Secondly, the incorporation of Ag/Cu nanoflakes offered a more cost-effective alternative, mitigating cost concerns while maintaining desirable conductivity levels, which could offer a more economically viable solution for LSP systems in aircraft structures.

Additionally, the choice of Ag/Cu nanoflakes is also influenced by their mechanical properties containing ductility and extremely high thermal conductivity of Ag are also advantageous. Ag/Cu nanoflakes typically have a high aspect ratio, which can enhance the coating's adhesion to the substrate and improve its surface mechanical strength, making it more resistant to delamination, cracking, wear, and other forms of damage. Furthermore, the optical microscope images of composite samples including Ag/Cu nanoflakes promote uniform dispersion within the coating matrix, ensuring consistent conductivity throughout the material. It's essential to ensure that the conductive filler can be uniformly dispersed within the epoxy resin without compromising its conductivity. The choice of Ag/Cu nanoflakes have been driven by their compatibility with epoxy carriers, allowing for the development of a coating system that maintains its conductivity and mechanical integrity over time. Overall, the selection of Ag/Cu nanoflakes as the conductive filler in the coating mixture in this study was motivated by their potential to offer synergistic electrical conductivity, cost-effectiveness, favorable mechanical properties, and compatibility with epoxy carriers. These characteristics make them a promising candidate for developing effective LSP systems for TPC aircraft structures, addressing the challenges associated with TPCs' weak electrical conductivity. In this study, the conductive spray-coated TPC panels were assessed for resistance to lightning strike damage according to the SAE ARP 5412B, SAE ARP 5414, and SAE ARP 5416 standards.

## Experiment

### Materials

For evaluating the lightning strike resistance of the spray-coated TPC, a substrate made of Toray TC1225 unidirectional tape, low-melt polyaryletherketone (LMPAEK)/CF (145 gsm) was purchased from Triumph Aerospace Structures. The TPC substrate comprised 16 layers of quasi-isotropic composite with dimensions of 20 × 20 inches (50.8 × 50.8 cm) with an average thickness of 2.0 mm, providing an appropriate platform for the lightning strike tests. The conductive nanomaterials used in this study were purchased from Technic, Inc. and consisted of two variations: SilCoflake 93–102 and SilCoflake 93–104. These nanomaterials comprised Ag/Cu nanoflakes with a ~ 5.0-micron planar and a thickness of about 100 nm. In addition, SilCoflake 93–102 had an Ag content of 20 wt.%, while SilCoflake 93–104 had an Ag content of 30 wt.%. The actual percentage of Ag content of each batch is tested and provided to the customer in the datasheets provided by Technic, Inc. The Ag/Cu nanoflakes were thoroughly washed and stored in ethanol before being used in the study. The study utilized a specific mixture of epoxy resin, consisting of Epon Resin 815C, a low-viscosity bisphenol-based epoxy resin containing n-butyl glycidyl ether with a viscosity range of 500–700 cP, and a curing agent called Epikure 3282, a modified aliphatic amine adducts with moderate viscosity of 2900–4900 cP, both of which were procured from Miller Stephenson. The Epon Resin 815C and curing agent Epikure 3282 were carefully blended in a 100:20.5 ratio, obtaining the perfect mixture for an epoxy resin. The surface was prepped with Sherwin Williams CM0481968, an epoxy primer, before being coated with the final layer, Sherwin Williams Y10942 White 7568 Topcoat, providing a smooth and durable finish.

### Methods

#### Surface measurements

Morphologies of conductive spray-coated TPC panels and sizes of used Ag/Cu nanoflakes were observed by Hirox RH2000 Optical Microscope. The electrical resistivity of the TPC panels was recorded by a High-precision four-point measurement device for milli-ohms range resistance, Hioki RM3548 Resistance Meter. The conductivity was obtained by calculating the electrical resistivity of four measurement points of the TPC panel for acquiring accurate values.

#### Conductive lightning strike protection coating

**Fig. 2:**
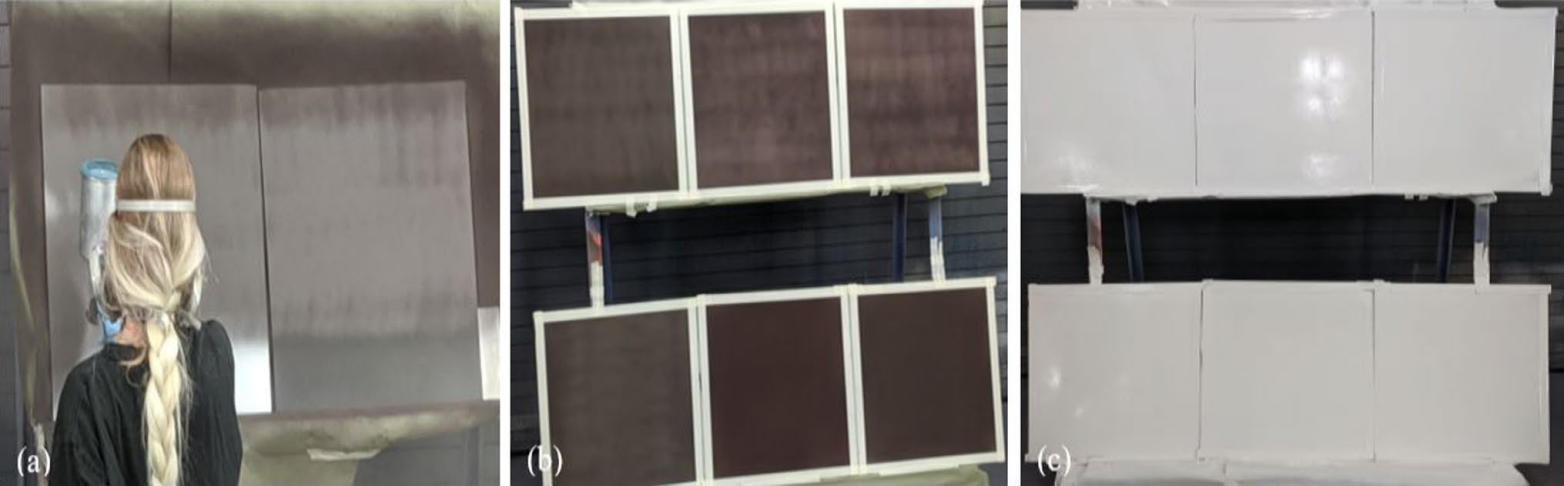
Visual representation images of the **a** two panels being spray coated with Ag30/Cu at loading of 55 wt.%: Panel on left spray-coated with HBN dielectric (20 wt.%) coat; **b** Six panels with conductive coatings; and **c** Six panels with topcoat paint

The process of creating conductive LSP coatings on TPCs involved blending an ethanol suspension of Ag/Cu nanoflakes into the epoxy mixture. The first step was to slowly incorporate the conductive filler into 5 g of solvent in a 25 mL beaker and then stir it with a magnetic stirrer at 250 rpm for 15 min. to achieve a well-mixed solution. Then, 5 g of epoxy resin was added to the solution and stirred for an additional 15 min. to thoroughly combine the ingredients. Then, the stirring speed was gradually increased based on the wt.% loading of the conductive filler in the mixture to ensure proper mixing of the conductive filler into the epoxy portion. Next, the 25 mL beaker containing the mixture was secured in a water bath inside a 1000 mL beaker with a 3D printed jig to prevent slipping off center during sonication. Each time, the mixture was sonicated using the Sonics VCX-130 PB Ultrasonic Processor with a 40% amplitude for a duration of 45 min. After sonication, the mixture was stirred again on the magnetic stirrer until a suitable spraying viscosity was reached. Finally, the conductive LSP coating solutions (~ 178 gsm), which had not been subjected to heat to prevent damage to the nanomaterials, were carefully taken off the magnetic stirrer and applied to the TPC panel via a high-volume low-pressure (HVLP) spray gun at room temperature. Six 20 in. × 20 in. (50.8 × 50.8 cm) panels were selected to assess the lightning strike resistance. The panels were first scuffed using 320-grit sandpaper to ensure the surface was prepared correctly. Afterward, they were thoroughly cleaned using isopropyl alcohol, and each panel was weighed individually. An aluminum plate was positioned between each panel to ensure that the thickness of the coating was measured accurately during each spray cycle. The thickness of the coatings was controlled by a spreader and measured using PosiTector 6000 Series coating thickness gauge. Conductive and dielectric coatings were oven cured at 85 °C for 18 h. and then applied to the six panels in the following portions:

Four panels, Panel 1, Panel 2, Panel 4, and Panel 5 were spray-coated with different prescribed levels of Ag20/Cu at loading of 55 wt.% (Panels 1 and 2) and Ag30/Cu at loading of 55 wt.% (Panels 4 and 5). Two panels (Panels 1 and 4) were equipped with hexagonal boron nitride (HBN) dielectric (20 wt.%) from these four panels. The thickness of the HBN dielectric (20 wt.%) coating was determined to be 75 µm. The other two panels (Panels 2 and 5) were left without HBN dielectric (20 wt.%) coating. Figure 2a shows the two panels with (Panel 4) and without (Panel 5) HBN dielectric (20 wt.%) were spray coated with Ag30/Cu at loading of 55 wt.%. Finally, the remaining two panels were spray-coated with Ag20/Cu at loading of 70 wt.% (Panel 3) and Ag30/Cu at loading of 70 wt.% (Panel 6). After applying the coating, the panels were allowed to cure at room temperature for 6 h. before being transferred to an industrial oven, where they were subjected to a temperature of 90 °C for 12 h. The thickness of the conductive coating was determined to be 75 µm. Figure 2b shows all six panels with borders masked after applying the conductive coat. Then, the final top coat paint was applied with a thickness of approximately 125–150 µm, as depicted in Fig. 2c [20, 68].

#### Lightning strike test equipment

**Fig. 3:**
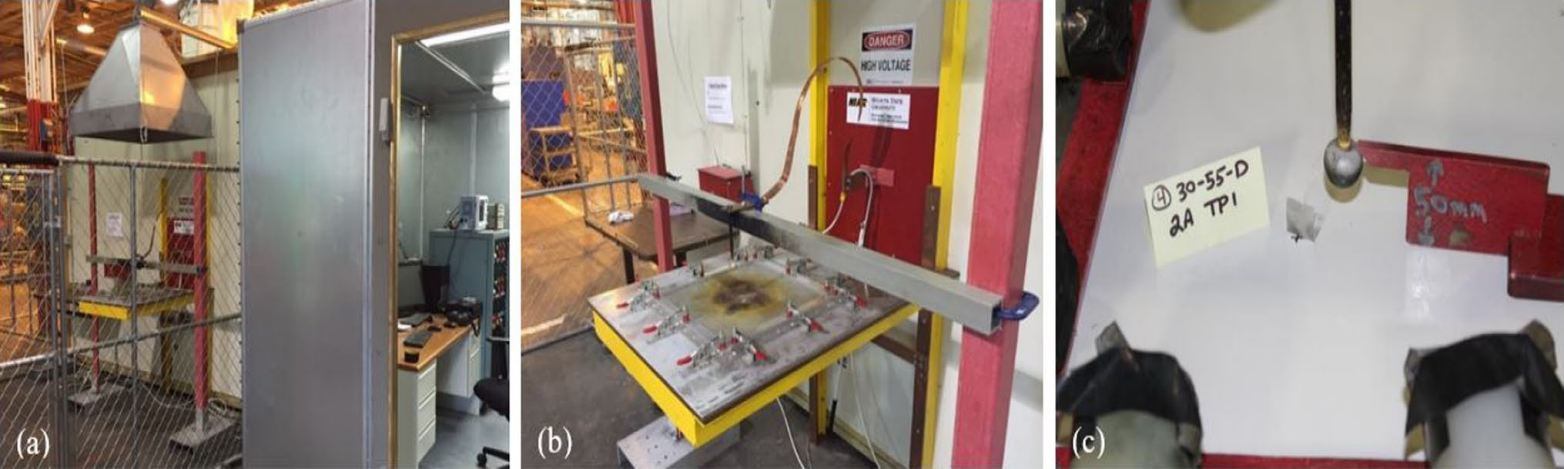
The images showing **a** DEL generator and test control room; **b** DEL test fixture with aluminum calibration test fixture; and **c** Aircraft zone 2A setup with 20 in. × 20 in. panel

According to the guidelines established in SAE ARP5416, Aircraft Lightning Test Method, lightning strike testing was performed on the TPC panels. This involved covering the panels with an aluminum-framed test fixture and placing a fiberglass plate on top. The aim of the procedure was to ensure that the current would be able to flow away from the TPC panel and toward the ground in any direction. To accomplish this, a diverting electrode was positioned 50 mm above the TPC panel, with an insulating ball fixed at the end of the electrode. In order to ensure that the end of the initiating wire was a few millimeters above the TPC panel, the wire was attached to a piece of Scotch tape. The tape was placed so that half of it was attached to the TPC panel, with the other half bent upwards, creating a hinge-like mechanism that allowed the end of the wire to project upwards from the TPC panel. Figure 3a displays the Direct Effects Lightning (DEL) generator and the test control room, and Fig. 3b depicts the DEL test fixture equipped with the aluminum calibration test fixture. The clamps that secure the TPC test panel are electrically insulated from the panel using dielectric sleeves. The test TPC panel is positioned and securely fastened onto the aluminum frame, which has a direct connection to the ground. Figure 3c displays the placement of the probe that is situated 50 mm above the surface of the test TPC panel. The probe is equipped with a dielectric ball to prevent direct electrical arc contact with the TPC panel, as specified in the SAE ARP5416 Aircraft Lightning Test Method [65].

#### Lightning strike test method

**Fig. 4:**
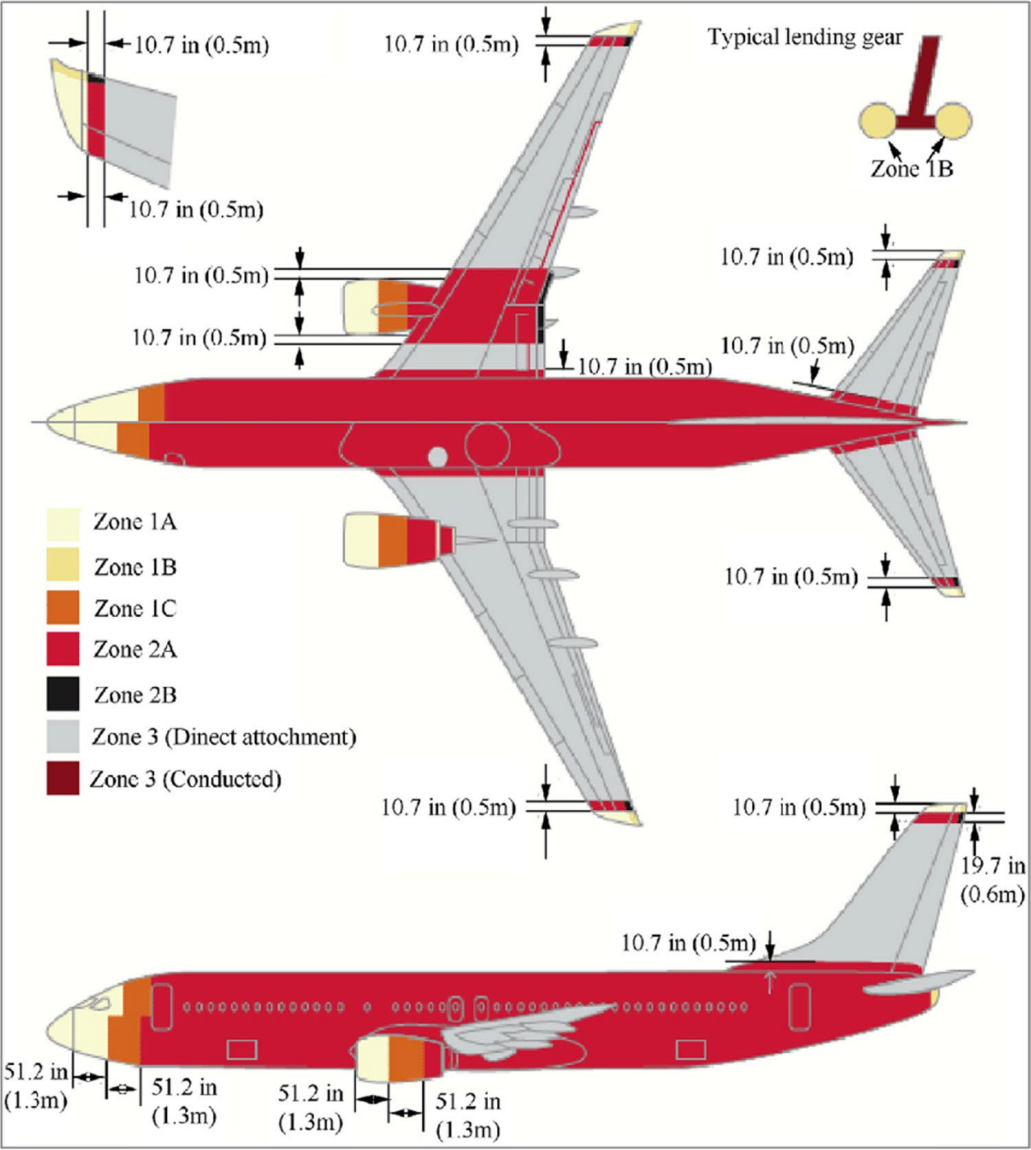
Various zones on an aircraft that are more susceptible to lightning strikes [69]

The lightning strike tests are aimed at assessing the impact of lightning strike currents on the spray-coated TPC panels and evaluating the LSP’s performance. Lightning strikes are not uniform and affect all parts of an aircraft depending on the location/zoning area. The strength and length of the currents that enter various surfaces of the aircraft can differ based on their positions. Specialized areas known as lightning strike zones have been defined to address these variations. Identifying these zones on a specific aircraft makes it possible to understand the lightning environment it is exposed to and determine the necessary protection requirements. The placement of these zones on any given aircraft is influenced by its physical design and usage patterns and can differ significantly from aircraft to aircraft. Zone 1, designated as the initial point of contact during a lightning strike, means that the extremities of an airplane should be considered part of the primary strike zone. The designations of Zone 1A and Zone 1B refer to the classification of different parts of an aircraft’s extremities. Specifically, the leading edges are within Zone 1A, while the trailing edges are within Zone 1B. The areas immediately following Zone 1A are referred to as Zone 2A, which is characterized as a zone in which lightning strikes are less likely to persist and cause damage as they are swept away more quickly. It is imperative to note that specific regions of aircraft surfaces are particularly susceptible to lightning strikes, characterized by a first return of reduced amplitude. These areas typically exhibit a low likelihood of sustaining a prolonged flash hang-on following lightning channel attachment, which extends from the nose of the aircraft to the point where Zone 1A ends. If lightning strikes an area behind Zone 2A, it is classified as Zone 2B or Zone 1B, depending on whether the initial attachment occurs. Any surface on the aircraft that is not part of zones 1A, 2A, 2B or is not likely to make direct contact with a lightning channel is considered to be within zone 3. The areas that fall under Zone 3 can be exposed to a significant amount of electrical energy during a lightning strike. As depicted in Fig. 4, the lightning strike zones of an airplane are clearly illustrated, and it is generally noted that for conventional airplane designs, the fuel tank is positioned within both Zone 2A and Zone 3, thus making it susceptible to being affected by the electrical energy carried by lightning strikes. The different zones on an aircraft susceptible to lightning strikes correspond to specific sets of current components. The lightning current components outlined in SAE ARP 5412B and presented in Table 1 are commonly used to examine the DEL through high current injection tests [66, 69–72].

**Table 1:** Lightning zone of aircraft and corresponding current components for direct effect lightning

Aircraft zone	Current components
Zone 1A	A, B, C*
Zone 1B	A, B, C, D
Zone 1C	A_h_, B, C*, D
Zone 2A	D, B, C*
Zone 2B	D, B, C
Zone 3	A, B, C, D

**Fig. 5:**
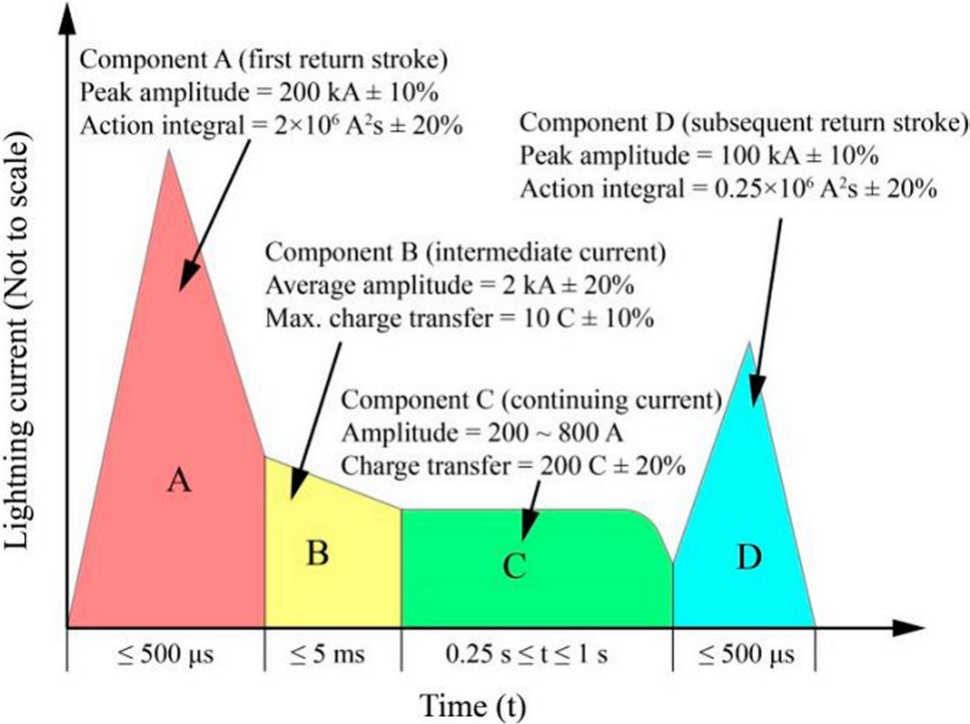
Standardized simulated lightning current waveforms [73]

In order to assess the direct effects of lightning strikes, four different current components (A, B, C, and D) are employed. However, in this study, the lightning strike test focuses on Aircraft Zone 2A, which requires the use of current components D, B, and C*, the specifications for which are outlined in Table 2, and the current–time curves for D, B, and C* waveforms can be seen in Fig. 5.

**Table 2:** Simulated lightning current waveforms per SAE ARP 5412B [66]

Component D	(Subsequent return stroke)
Peak amplitude	100 kA (± 10%)
Action integral	0.25 × 10^6^ A^2^s (± 20%)
Time duration	500 µs
Component B	(Intermediate current)
Average amplitude	2 kA (± 10%)
Max. charge transfer	10 Coulombs (± 10%)
Time duration	5 ms
Component C*	(Continuing current)
Amplitude	200–800 A
Charge transfer	200 Coulombs (± 20%)
Time duration	0.25 to 1 s
Component A	(First return stroke)
Peak amplitude	200 kA (± 10%)
Action integral	2 × 10^6^ A^2^s (± 20%)
Time duration	500 µs

Figure 5 shows the four current components, their electrical characteristics, and their expected effects on aircraft structures [73]. The four components, A, B, C, and D are used to represent the lightning strike waveform, which can be broken down into two distinct categories. The first category, comprised of components A and D, is characterized by having a short duration, small-transferred charge, high action integral, and high current amplitude. The second category, composed of components B and C, is defined by having a longer duration, large-transferred charge, low action integral, and low current amplitude. They can be applied individually or in combination with one or more other components during a single test. The first component, referred to as component A, is meant to simulate the initial high peak current during a lightning strike. It has a peak current amplitude of 200 kA, and the total time duration of this component is limited and does not exceed a certain threshold. The second component, known as component B, represents the intermediate current flow. This component has an average current amplitude of 2 kA and a maximum duration of 5 ms. Component C, also known as the continuing current, is responsible for transferring a charge of 200 coulombs over a period that falls between 0.25 and 1 s. On the other hand, Component D also referred to as the restrike current, is characterized by its peak amplitude of 100 kA. This component can be unidirectional or oscillatory, and its total time duration should not exceed a certain limit [66].

## Results and discussion

### Optical microscope results

**Fig. 6:**
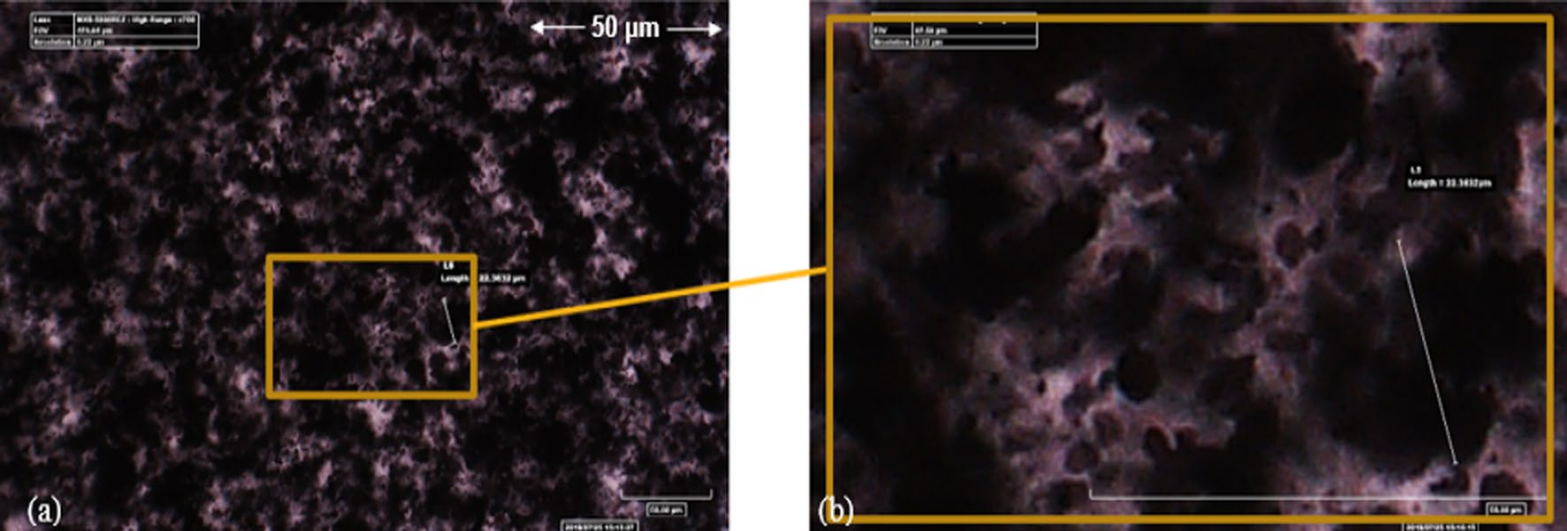
**a** Conductive filler, Ag30/Cu nanoflakes with a silver content of 30 wt.% in an epoxy matrix; **b** with more detail scale bar = 50 µm

The utilization of an optical microscope to examine the initial samples was a beneficial approach to evaluate the efficiency of dispersion and surface morphologies for conductive nanofillers, especially for loadings of up to 30 wt.%. It was observed that the resin system allowed sufficient transmission of light. The resin system, after the curing agent was added and in its cured state, had a reddish-brown translucent appearance. It was possible to observe the agglomeration easily, and in the instance of Ag/Cu, individual nanoflakes could be distinguished. In Fig. 6, a detailed illustration is presented, which shows the absence of a connected network of conductive Ag30/Cu nanoflakes. Figure 6a presents a networked appearance, while the other image illustrates the presence of gaps between the filler particles, resulting in a lack of a continuous conductive path at loading of 30 wt.%. The blurry and sharp-edged masses in the image suggest that they are located on separate focal planes. The particle distributions of the various Ag30/Cu nanoflakes were found to be uniform, with no obvious signs of aggregation. Furthermore, the silver particles within the composites were observed to be spherical, which can have important implications for their conductivity and other properties.

### Resistance of lightning strike protection systems

**Fig. 7:**
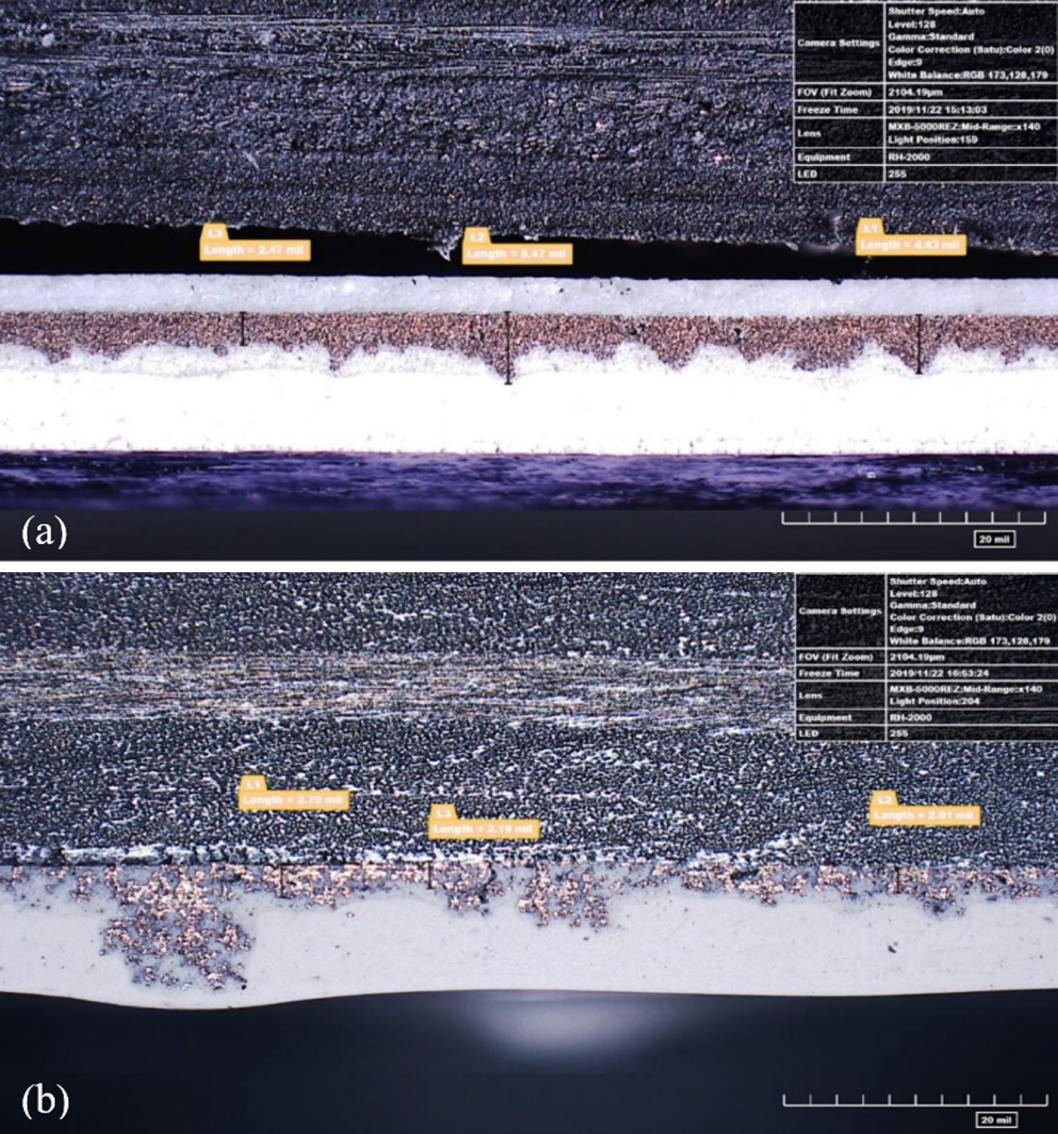
Cross-sectional micrographs of **a** Panel 2 (Ag20/Cu at loading of 55 wt.%); and **b** Panel 3 (Ag20/Cu at loading of 70 wt.%)

During resistance measurement testing, both Ag20/Cu and Ag30/Cu showed resistance levels of 0.6 Ω/sq and 0.8 Ω/sq, respectively. However, when the same materials were tested on spray-coated lightning strike test panels, the average resistance levels increased to 1.0 Ω/sq. The Panels 3 and 6 had a rough surface, which was noticeably different from the texture at loading of 55 wt.% TPC panels. Figure 2 shows the cause for the rough surface. The experiments showed that when a high amount of conductive filler was used in combination with an HVLP spray gun, it led to a dry spray. This dry spray prevented the flakes from staying smooth on the panel's surface. Figure 7b provides an evident illustration of Panel 3's thinly spread and loosely packed conductive layer, which contrasts with Panel 2's conductive layer in Fig. 7a. The loose packing of the conductive layer is likely responsible for the higher electrical surface resistance.

### Lightning strike test results

**Fig. 8:**
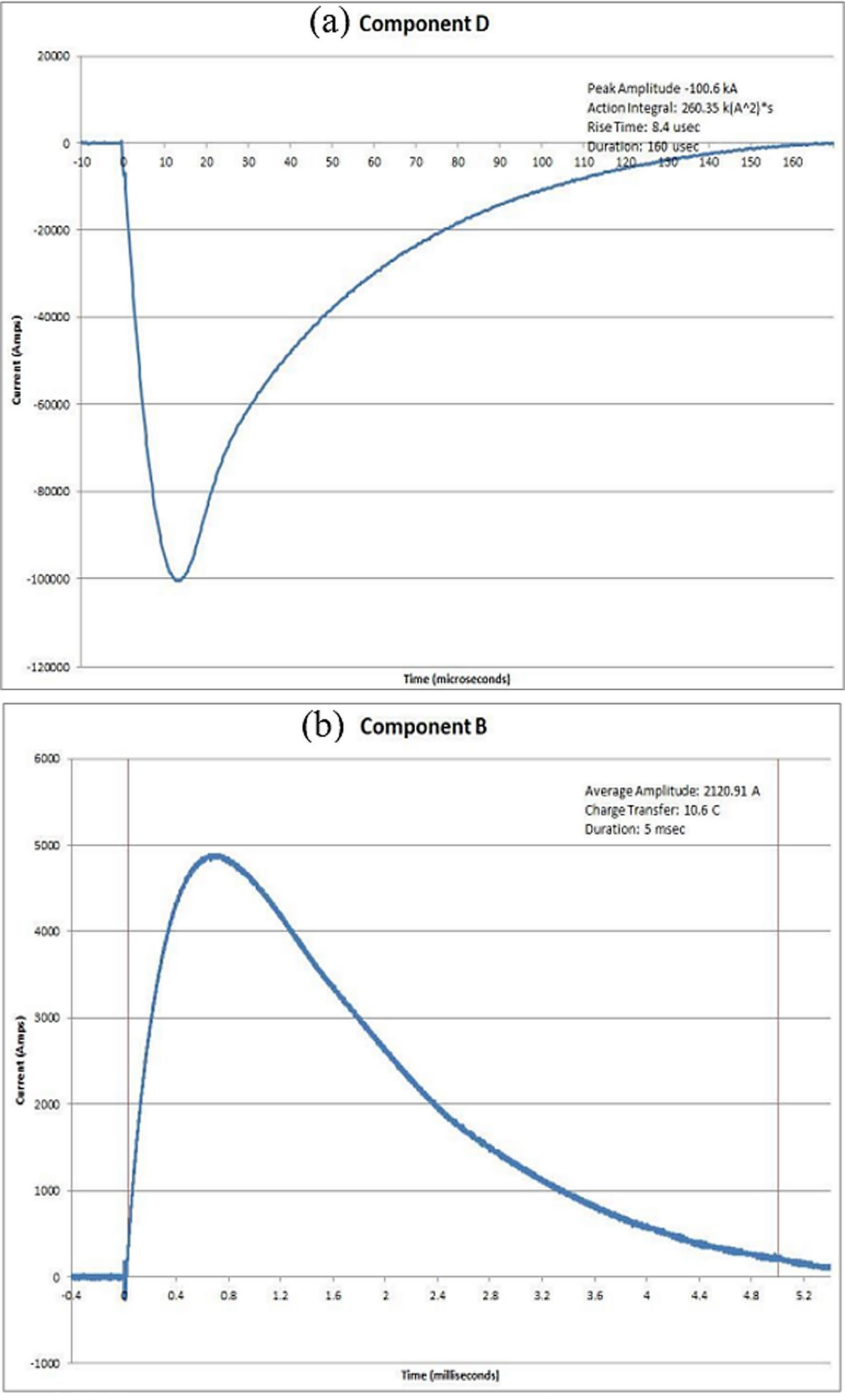

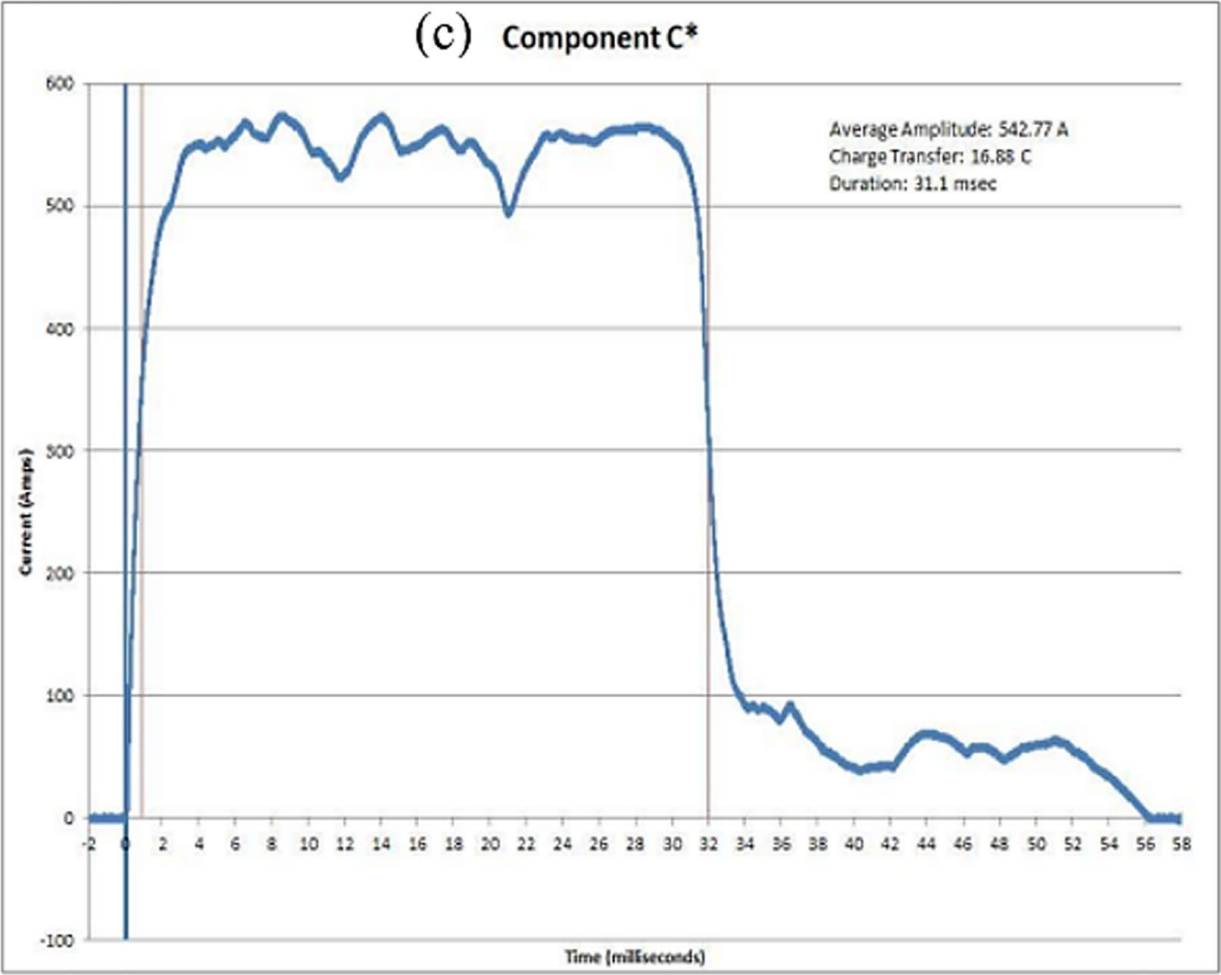
Waveforms and parameters of the various lightning current components, **a** lightning component D; **b** lightning component B; and **c** lightning component C*, with each component exhibiting distinct characteristics during the strike event

The results of a lightning strike test are presented in this section. The test parameters met the criteria outlined in SAE ARP 5412B, which outlines the requirements for high-current waveform testing. All the data for the DEL generator test waveform components, including components D, B, and C*, are obtained and listed in Table 3. Figure 8a–c provide graphical representations of the calibration tests that were conducted on an aluminum test panel. These graphs demonstrate the accuracy of the lightning strike testing performed. The results of the TPC protected against lightning strikes were as anticipated, corresponding to the fibers not affected by the lightning current in the first layer of the unidirectional ply. In the scenario involving the application of conductive fillers, it was observed that the impacted area was slightly larger due to the burn caused by the conductive surface coating. However, the protective effect of the TPC structure is noticeable, as it appears to be preserved in depth. Some vital criteria were utilized to assess the damage caused by a lightning strike on the TPC panels, including fiber damage on the panel, delamination on the panel, and holes that penetrated the panel.

**Table 3:** Parameters of the lightning component waveforms in various test modes

EUT	Test Point	Compon ents	Component A'D				Component B			Component C.-'C"		
			Peak Amplitud a(kA)	*M*	Rise Time (usee)	Duration (usee)	Average Amplitud e(kA)	Charge Transfer (Coulorn bs)	Duration (msec)	Average Amplitud •(A)	Charge Transfer (Coulom bs)	Duration (msec)
Aluminum Panel	Wavefor m Verificati on	D, B, C*	− 100.6	2.60E + 05	8.4	160	2.12E + 03	10.6	5	542.77	1688	31.1
Panel 1 20–55-D	TP 1	D, B, C*	− 103.53	2.40E + 05	8.2	108	2.05E + 03	10.25	5	403.61	22 53	55.82
Panel 1 20–55-D	TP 2	D, B, C*	− 102.8	2.31 E + 05	8.4	103.4	2.05E + 03	10.24	5	401.48	22.89	57.01
Panel 2 20–55	TP 1	D. B, C*	− 102.6	2.28E + 05	8.2	106	2.05E + 03	10.26	5	418.28	22 38	53.5
Panel 2 20–55	TP 2	D, B. C*	− 102.93	2.32E + 05	8.4	105.2	204E + 03	10.21	5	− 0.67	0	0
Panel 3 20–70	TP 1	D, B. C*	− 104.27	2.47E + 05	8.2	117.8	2.06E + 03	10.29	5	405.48	22 84	56.32
Panel 3 20–70	TP 2	D, B. C*	− 104.53	2.48E + 05	8.2	119	2.04E + 03	10.2	5	391.59	23.47	59.93
Panel 4 30–55-D	TP 1	D, B. C*	− 103.8	2.41 E + 05	8.4	110.4	2.01E + 03	10.06	5	414.25	22.04	53.2
Panel 4 30–55-D	TP 2	D, B. C*	− 103.67	2.42E + 05	8.4	111.8	2.04E + 03	10.19	5	387 28	22.97	59.32
Panel 5 30–55	TP 1	D. B. C*	− 103.8	244E + 05	8.4	116	203E + 03	10.13	5	430.29	21.1	49.03
Panel 5 30–55	TP 2	D, B, C*	− 104.07	2.42E + 05	8.4	113.6	206E + 03	10.3	5	429.57	21.16	49.26
Panel 6 30–70	TP 1	D, B, C*	− 104.73	2.51 E + 05	8.4	120.6	2.04E + 03	10.22	5	434.55	21.11	48.58
Panel 6 30–70	TP 2	D, B. C*	− 104.67	2.51 E + 05	8.4	121.2	2.05E + 03	10.27	5	428.53	21.39	49.91

#### Lightning damage definitions

*Fiber damage on the composite panel* The measurement of exterior fiber damage on the TPC panel is the side that receives the lightning current and is also referred to as the front of the panel. The length of broken fibers between their attachment endpoints and the panel surface in the longitudinal direction determines the fiber damage. This length also represents most of the resin loss area, and the width of all broken fibers is measured in the transverse direction. If there is more than one isolated area of broken fibers, each area is calculated separately, then the total is determined. It is uncommon for fiber damage in the deeper plies to surpass the dimensions of the damage to the outer ply, excluding delamination. The interior fiber damage is located on the bare side of the panel that faces away from the point of attachment of the lightning current. It is sometimes referred to as the back of the panel. This damage is measured by using the length of broken fibers between the attachment endpoints to the inner surface of the panel in the longitudinal direction. The value obtained from this measurement represents most of the resin loss area. The width of all broken or damaged fibers along the transverse direction was also measured. If there are multiple distinct areas of damaged fibers as a result of a split attachment or some other abnormal situation, each area would be measured separately and then combined to find the total area of damage. The fiber damage area on the front of the panels with the Ag30/Cu conductive filler was more significant than that for Ag20/Cu. The only instance where fiber damage was observed on the back of the panel was test point (TP)1 for Panel 2 with the Ag20/Cu conductive filler and no dielectric, as listed in Table 4.

**Table 4:** Comparison between each panel’s fiber damages

Panel ID	Ag/Cu	Wt%	Dielectric Layer	Test Pt	Fiber Damage: Front			Fiber Damage: Back		
					Longitudin	Transverse	Area (sq/in]	Longitudin	Transverse	Area (sq/in
1	20	55	D	1	2.25	1.75	3.9375	0	0	0
1	20	55	D	2	2	1.5	3	0	0	0
4	30	55	D	1	2	1.25	2.5	0	0	0
4	30	55	D	1	0.25	1.5	0.375	0	0	0
4	30	55	D		2.25	2.5	5.625	0	0	0
2	20	55		1	3	2.25	6.75	0.25	0.25	0.0625
2	20	55		1	0.125	0.5	0.0625	0	0	0
2	20	55		2	2.5	2.25	5.625	0	0	0
2	20	55			0.25	0.25	0.0625	0	0	0
5	30	55		1	3	2.5	7.5	0	0	c
5	30	55		1	0	0	0	0	0	0
5	30	55		2	2.25	3	6.75	0	0	0
3	20	70		1	2.75	3	8.25	0	0	0
3	20	70		2	2.75	3.5	9.625	0	0	0
6	30	70		1	4.25	3.25	13.8125	0	0	0
6	30	70		2	3	3.5	10.5	0	0	0

*Delamination on the composite panel* The exterior delamination of the panel is evaluated by conducting a tap test which involves tapping the exterior surface of the panel using a heavy steel washer. The test is to listen for a change in the tone or deadening of the usual ringing response of the panel as the washer approaches the damaged area (for a rough analysis). The exterior delamination is determined by the extent to which the tone or ringing response of the panel changes. The test is performed both in the direction of the outer ply’s longitudinal fibers and in the transverse direction. The measurement provides insight into the detachment of one ply from another, with the method being particularly effective in detecting delamination near the surface ply. Additionally, exterior delamination can also detect loss of resin or damage to fibers on the surface layer, as opposed to just the separation between two plies without any damage to the fibers, which can occur between any two plies. The measurement of interior delamination is conducted through the same approach as that of exterior delamination, with the only difference being that it is carried out by tapping on the panels' interior. The outcome of this measurement represents the separation of one ply from the next, and it is more prone to be sensitive to the plies located close to the inner surface layer. Table 5 provides the data for measurement of delamination, which has been averaged based on the TP 1 and TP2 values from Panel 1 to Panel 6 on the front and back of each panel.

**Table 5:** Comparison between each panel’s delamination

Panel ID	Ag/Cu	Wt°/o	Dielectric Layer	Test Pt	Delamination: Front			Delamination: Back		
					Long	Trans	Area (sq/in)	Longitudin	Transverse	Area (sq/in;
1	20	55	D	1	2.5	2.25	5.625	0		0
1	20	55	D	2	2.5	2.5	6.25	3	2.75	8.25
4	30	55	D	1	3	3.25	9.75	0	0	0
4	30	55	D	1	3	1.75	5.25	0	0	0
4	30	55	D	2	3.25	2.75	8.9375	1.25	0.75	0.9375
2	20	55		1	3.25	2.5	8.125	11.5	1.5	17.25
2	20	55		1	1	1	1	0	0	0
2	20	55		2	3.25	4	13	1	2.75	2.75
2	20	55		2	0	0	0	0	0	
5	30	55		1	4	2.75	11	0	0	0
5	30	55		1	0	0	0	0	0	0
5	30	55		2	3.75	3	11.25	0.25	0.5	0.125
3	20	70		1	3.75	5	18.75	0	0	0
3	20	70		2	3	3.2	9.6	0	0	0
6	30	70		1	4,25	3.25	13.8125	0	0	0
6	30	70		2	3	3.5	10.5	0	0	0

*Hole through the composite panels* A hole through the panel is considered the most severe damage caused by a lightning strike on the TPC panel. This measurement refers to the hole size that completely penetrates the panel in longitudinal and transverse directions. The primary objective of the LSP system is to prevent this type of damage from occurring while flying. Additionally, several direct comparisons are discussed in order to determine the significance of each factor, which will aid in identifying the most effective and efficient configuration of these factors.

#### Inspection of lightning damage on composites

**Fig. 9:**
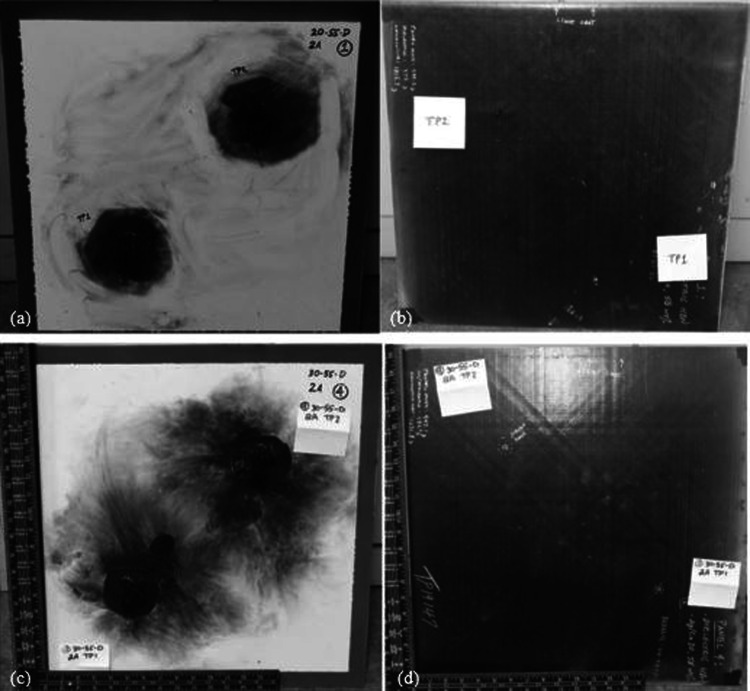
The after lightning strike images of **a** front of Panel 1 (Ag20/Cu at loading of 55 wt.% with HBN dielectric (20 wt.%)); **b** back of Panel 1; **c** front of Panel 4 (Ag30/Cu at loading of 55 wt.% with HBN dielectric (20 wt.%)); and **d** back of Panel 4

*Comparisons of silver contents* The initial stage of analysis following a lightning strike test involves a visual inspection. This inspection allows for the identification of the regions that have been affected by the lightning current. When the lightning struck, an intense and blindingly bright white light was emitted from the arc, while a fiery red flame was produced from the burning TPC panels. Additionally, the TPC panels revealed the presence of burst fibers as a result of the strike. It was observed that the degraded resin had produced a thick, dark plume of black smoke. Three panels have been designed with Ag20/Cu conductive nanofillers in an epoxy matrix, and three others have been designed with Ag30/Cu conductive nanofillers in the same epoxy matrix to compare the silver coating on copper nanoflakes. This allows a direct comparison between the content of silver on the copper nanoflakes in the panels. The target thickness for the conductive filler coatings for all panels was established at 75 µm and was achieved through a spray-coating process. Panel 1 and Panel 4 panels are made up of 55 wt.% nanofiller and feature a dielectric layer with a thickness range of 50 to 75 µm of HBN dielectric (20 wt.%). Figure 9 displays the after lightning strike images of the front and back of Panels 1 and 4, with the front of Panel 1 in Fig. 9a and the back in Fig. 9b. Similarly, Fig. 9c illustrates the after lightning strike images of the front of Panel 4 and the back in Fig. 9d.

**Fig. 10:**
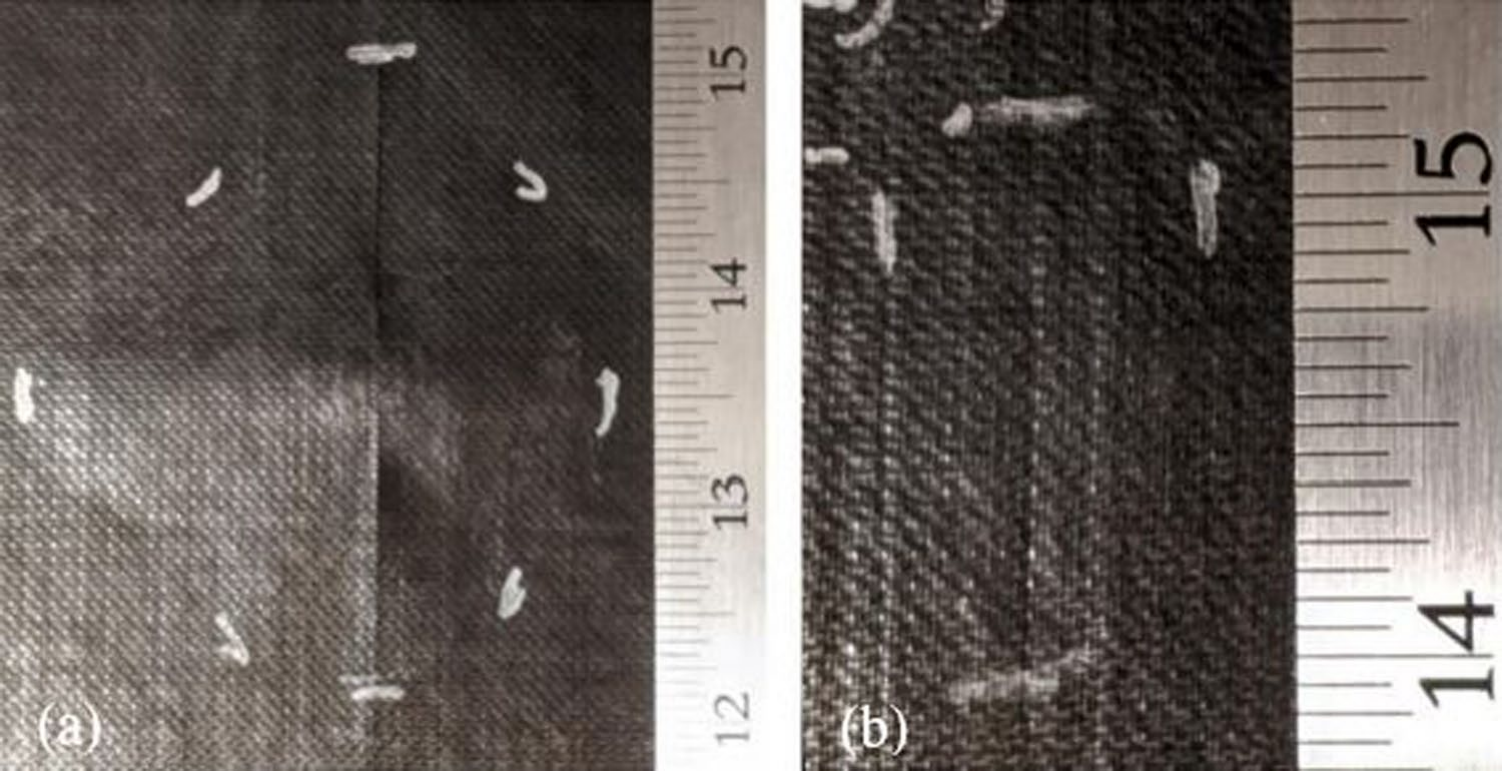
The after lightning strike detail of **a** Panel 1 (Ag20/Cu at loading of 55 wt.% with HBN dielectric (20 wt.%)), TP2; and **b** Panel 4 (Ag30/Cu at loading of 55 wt.% with HBN dielectric (20 wt.%)), TP2. A silver marker has been used to highlight the interior delamination for better clarity

The test results from Table 5 show that the average delamination value of Panel 1 is 5.93 in^2^ (38.26 cm^2^) while that of Panel 4 is 11.97 in^2^ (77.23 cm^2^). As depicted in Fig. 9c, the after lightning strike image reveals that Panel 4, TP1 has a distinct damaged region as well as the primary point of attachment. The delamination area in Panel 1, measured by TP2 on the back. This is significantly larger than that of Panel 4, TP2, by almost 900%, as depicted in Fig. 10. Specifically, the degree of damage in Panel 1 was more severe compared to Panel 4, even though they had similar types of damage. No interior delamination is observed in TP1 of Panel 1 and Panel 4.

**Fig. 11:**
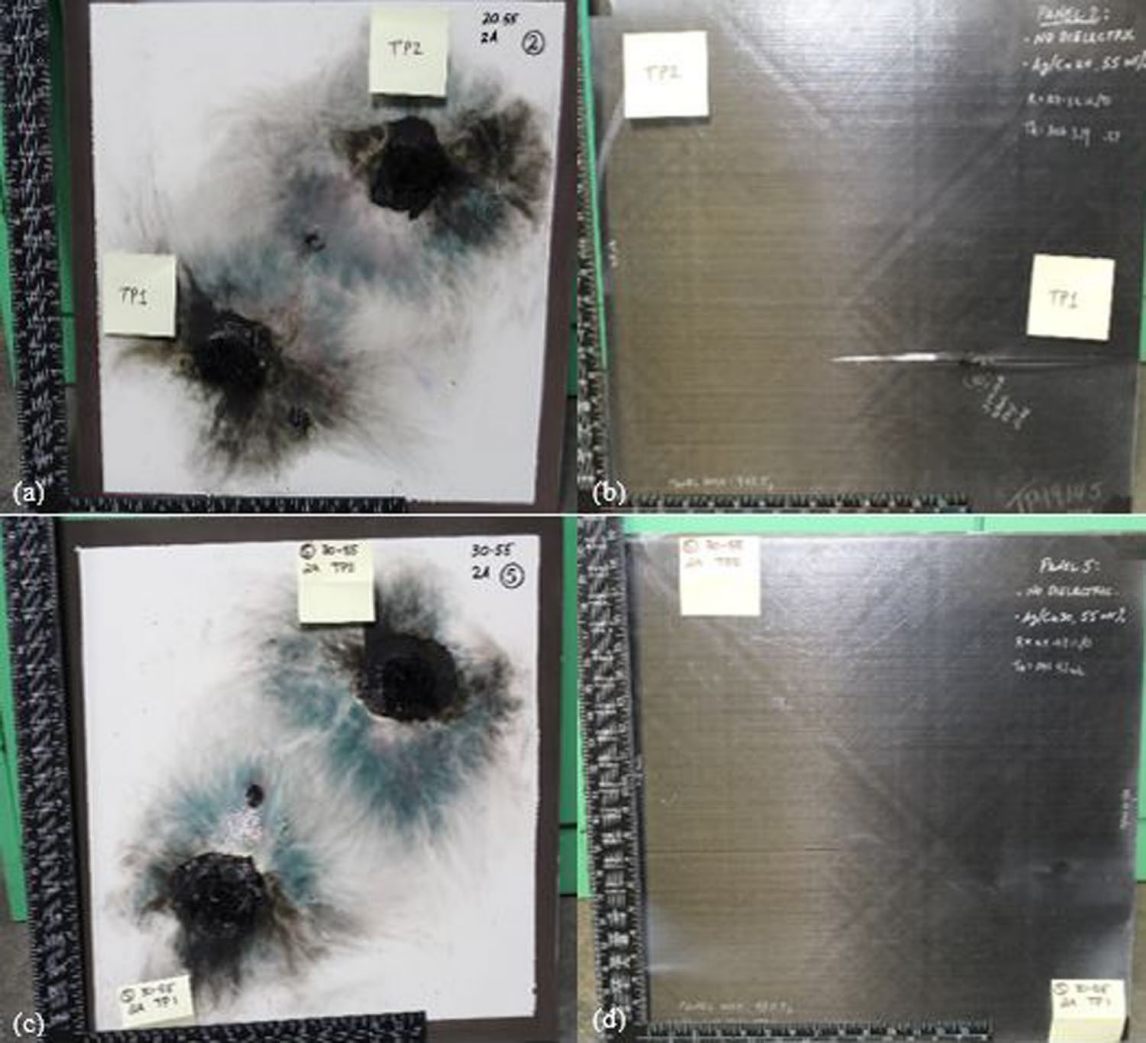
The after lightning strike images of **a** front of Panel 2 (Ag20/Cu at loading of 55 wt.%); **b** back of Panel 2; **c** front of Panel 5 (Ag30/Cu at loading of 55 wt.%); and **d** back of Panel 5

The delamination test results on Panel 2, which is made of conductive Ag20/Cu at loading of 55 wt.% nanofiller and without dielectric, revealed an average of 4.6 in^2^ (29.68 cm^2^) of delamination on the exterior and a significantly higher amount of 17.25 in^2^ (111.29 cm^2^) of delamination on the interior at TP1. At the same time, TP2 had 2.75 in^2^ (17.74 cm^2^) of interior delamination. On the other hand, Panel 5, made of conductive Ag30/Cu, has the same filler content as Panel 2 and, without dielectric, showed an average of 11.1 in^2^ (71.61 cm^2^) of exterior delamination and only 0.94 in^2^ (6.06 cm^2^) of interior delamination at TP2, with no delamination present at TP1. Figure 11 displays the post-test images of the front and back of Panels 2 and 5, with the front of Panel 2 in Fig. 11a and the back in Fig. 11b. Similarly, Fig. 11c displays the after lightning strike test of the front of Panel 5 and the back in Fig. 11d. The trend of larger delamination on the front panels was observed at loading of 55 wt.% panels with an HBN dielectric (20 wt.%) and loading of 70 wt.% panels. This disparity between Panel 4 having more significant exterior delamination and less interior delamination compared to Panel 1 can be explained by the superior ability of the Ag30/Cu nanofiller to distribute the heat energy generated by the lightning current more evenly over the top of the panel, thereby reducing the temperature increase. The flammability of the resin incorporated with the conductive particles might flame more. However, cause less damage to the composite panels. Investigations into the root causes of delamination have identified that the deformation of the panel is also mainly due to the sudden, explosive vaporization of the LSP and composite materials. Additionally, it makes sense that if the polymer layer between the plies maintains its structure, more energy will be needed to break the laminate apart.

In contrast, if the polymer in the LM PAEK is either melted or vaporized, then a lesser amount of energy may be needed to separate the layers. However, it is worth further examining if a thermoplastic polymer matrix within its glass transition temperature region operates as an exception. The high current density is commonly associated with lightning channels that can generate resistive heat, which may affect the temperature of the LSP system and the polymer’s rise above their pyrolization temperature instantly. This is due to the intense energy being released in a small area. If the current can be dispersed instantly over the panel's surface by a circuit with low resistance, it means that the TPC is less likely to reach its vaporization temperature since the current's energy is not as focused on the material, and less Joule heat is generated. Furthermore, when a relatively minor amount of material is instantly vaporized, including the LSP, there is also a lesser degree of explosive force to cause the separation of plies. Fiber breakage happens when the combined explosive forces resulting from air breakdown, shear forces, shock waves, resin vaporization, and LSP vaporization surpass the tensile strength of the fiber bundles.

**Fig. 12:**
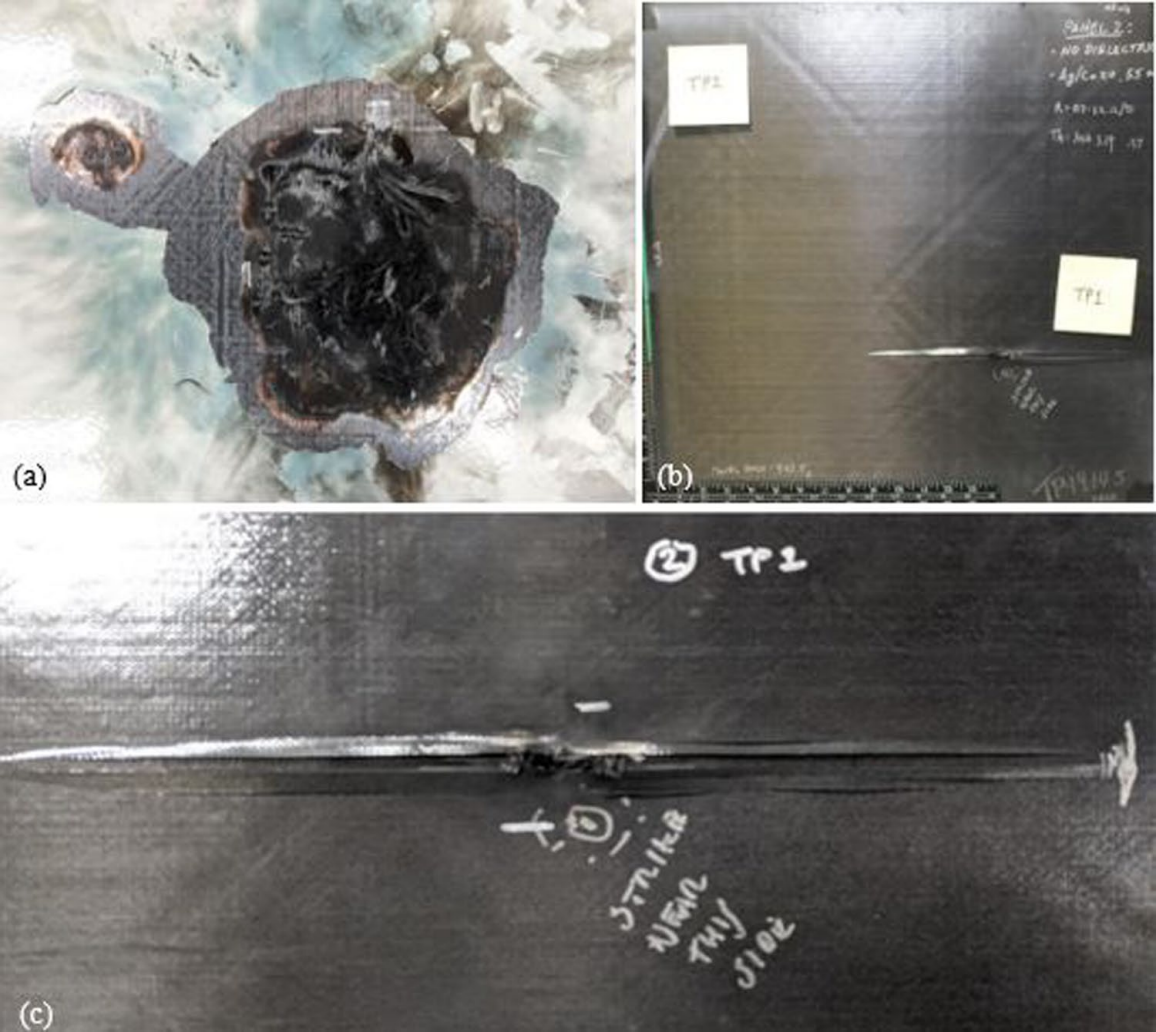
**a** Panel 2 (Ag20/Cu at loading of 55 wt.%), TP1, the central area of damage and an island with less depth of damage; **b** The back of Panel 2 highlights the fiber damage and delamination evident at TP1. No interior damage was observed at TP2, and **c** A close-up visualization of Panel 2 reveals an interior delamination as well as a hole

When it comes to fiber breakages, loss of resin is always seen to be connected and is typically a requirement for any damage. The LM PAEK polymer is pyrolyzed at a temperature above 600 °C, which is well below the pyrolysis temperature of the carbon fibers. In this study, the measurement of fiber damage was determined when the fibers detached from the laminate, resulting in resin loss. It’s important to note that the measurement of resin loss is included in this evaluation, but it is possible that in some instances, the loss of resin could surpass the extent of fiber breakage even though the fibers remain adhered to the polymer layer of the next lower ply. Nevertheless, the damage patterns observed in this study were consistent and uniform, with the extent of resin damage being equal to the degree of fiber detachment, as evident in Panel 2, TP1, as depicted in Fig. 12a. When a lightning strike occurs, the massive electrical current generates a strong electromagnetic field that causes the air around it to become ionized. As a result, the pressure in the immediate vicinity of the lightning strike zone increases significantly, leading to explosion of the vaporized lightning protection layer, resin, carbon fiber cracking, exfoliation of the resin matrix, and other forms of damage. The explosion of the lightning protection layer of TPC structures represents a significant factor contributing to mechanical damage. This explosion is primarily attributed to the rapid vaporization of the metallic mesh constituting the lightning protection layer, a consequence of Joule resistive heating. Notably, the potential influences of additional factors such as direct heat from the plasma channel and the vaporization of the matrix material of the lightning protection layer (epoxy resin) or the paint have not yet been fully explored for in existing analyses.

The fiber damage area on the front of the panels with the Ag30/Cu conductive nanofiller was more significant than that for Ag20/Cu conductive nanofiller, just as it was for the delamination area. As mentioned in Table 4, the only instance where fiber damage was observed on the back of the panel was TP1 for Panel 2 with the Ag20/Cu conductive nanofiller and no dielectric, with an area of only 0.0625 in^2^ (0.40 cm^2^). Moreover, TP1 is also notable for exhibiting the largest interior longitudinal delamination of 11.5 inches (29.21 cm), as depicted in Fig. 12b. The results of the TP2 on Panel 2 were consistent with the outcomes of the other panels, but it still incurred the third largest interior delamination. It’s worth noting that none of the other panels exhibited any fiber damage on the interior. Additionally, Panel 2’s TP1 was unique in that because it was the only panel that had a hole thoroughly drilled through it. This can be observed in Fig. 12c. Although the hole was only a small size of 0.03125 in^2^ (0.20 cm^2^), it is considered the most severe type of damage in terms of appearance. Due to the location where it is located on an aircraft, it can potentially result in the ignition of fuel vapors or the malfunctioning of electronic systems. The results differ between TP1 and TP2 due to variations in the thickness of the paint applied on the panel. When the paint is thicker, it can better contain the arc root, which results in the concentration of the energy from the lightning strike. The presence of thicker paint may also help to confine the vaporized LSP and resin, leading to a more intense percussive deformation of the panel. It has been widely recognized that damage tends to increase with an increase in paint thickness. When the LSP spray coated-TPC panel is struck by lightning, the electrical current flows within the TPC structure and keeps almost the entire lightning current in the more conductive and, less resistant. Given that the objective of using LSP is to limit the depth of damage, the presence of Ag30/Cu conductive filler appears to have been more effective in protecting the TPC panel than Ag20/Cu conductive filler.

**Fig. 13:**
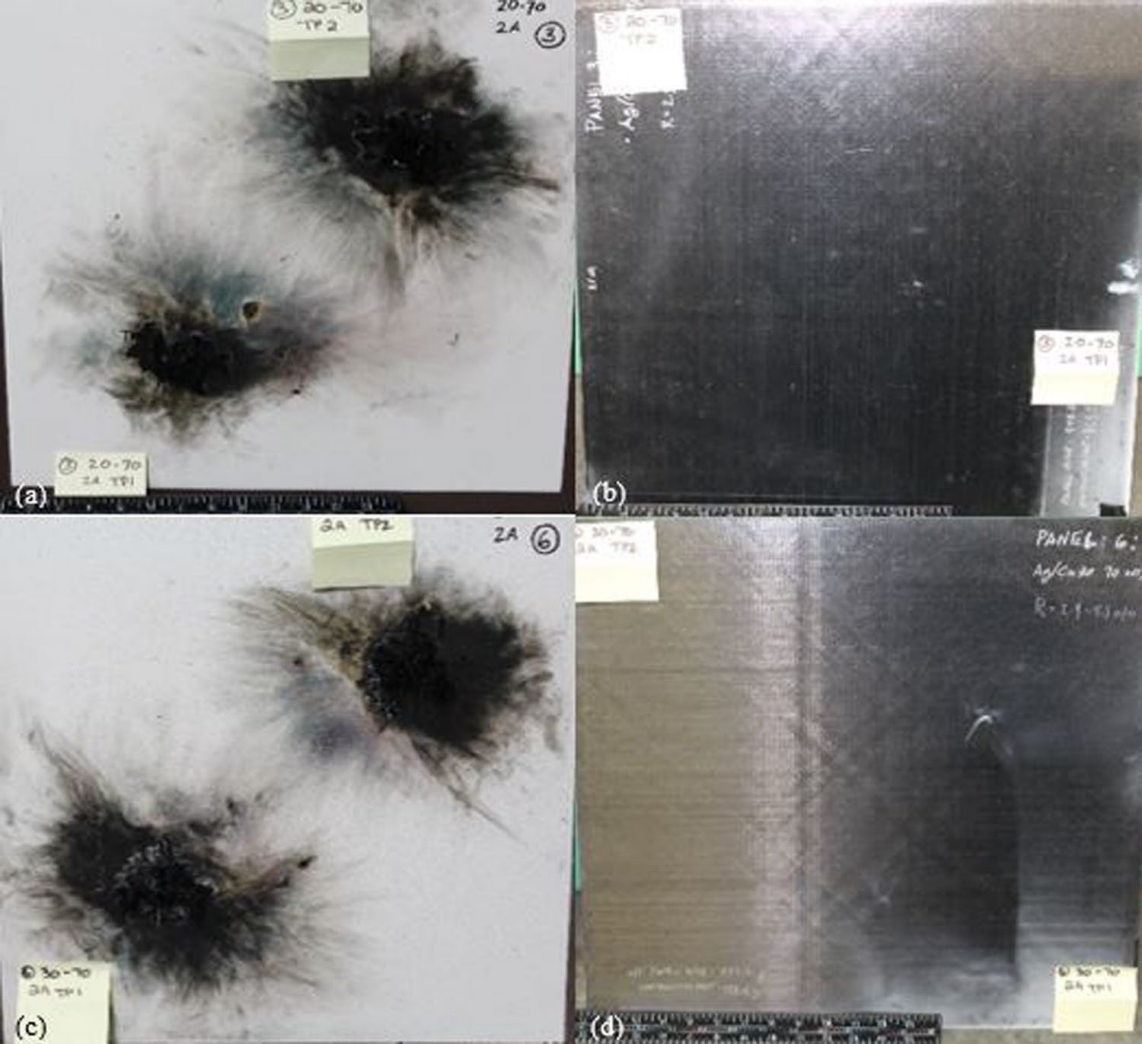
The after lightning strike images of **a** front of Panel 3 (Ag20/Cu at loading of 70 wt.%); **b** back of Panel 3; **c** front of Panel 6 (Ag30/Cu at loading of 70 wt.%); and **d** back of Panel 6

Figure 13 demonstrates the after lightning strike images of the front and back of Panels 3 and 6, with the front of Panel 3 in Fig. 13a and the back in Fig. 13b. Correspondingly, Fig. 13c shows the after lightning strike images of the front of Panel 6 and the back in Fig. 13d. Panel 6 exhibits less visual damage compared to Panel 5, as some of its fibers have been lost, and the edges of the damage appear curly while the cracking fibers are loose. This difference in damage is likely due to the fact that a portion of the lightning strike energy was conducted by the TPC panels, causing less damage to Panel 6. This observation supports the idea that using a conductive filler as a protective layer can effectively reduce the damage caused by lightning strikes to TPC panels. There were only a few fiber damages in the central damage zone of Panel 5, and the surface glossiness disappeared. The damaged area on Panel 5 was the largest compared to the damage on Panel 6. However, the damage depth of Panel 5 was significantly lower. This can be attributed to the fact that the re-solidified silver particles fixed the Ag/Cu layer on the TPC matrix surface, thus increasing the time needed to consume the lightning strike energy.

*Comparisons of nanofiller contents* The average mass of the Ag/Cu conductive nanofiller in TPC panels at loading of 55 wt.% is 25.2 g, whereas the average mass in TPC panels at loading of 70 wt.% is 15.3 g, which reduces the amount of conductive nanofiller used by 40%. However, this reduction in the conductive nanofiller mass does not fully explain the improved results seen in TPC panels at loading of 70 wt.%. The information presented in Table 6 highlights the larger areas of front-side delamination. This increase in size is because the plasma channel at the arc root tends to expand faster with more resistive protection measures, as the main part of the lightning current flows at the edge of the arc root (the current density within the arc root is much lower than that at the edge of the arc root), which can be attributed to the improved energy dispersion, as the interior damage observed was less extensive compared to similar panels. This relationship between improved surface energy dispersion and reduced damage through the depth is further established by the data listed in Table 7, which provides information on fiber damage. This result supports the notion that better energy dispersion leads to less damage, thus confirming the correlation between the two. By calculating the average of two TPs for each panel, it was found that the damage areas for the Panel 3 (Ag20/Cu at loading of 70 wt.%) were 65% larger, while the damage areas for the Panel 6 (Ag30/Cu at loading of 70 wt.%) were 41% larger. Furthermore, none of the TPC panels at loading of 70 wt.% displayed any fiber damage on the back side of the panels. However, visually, both (Ag20/Cu and Ag30/Cu) TPC panels at loading of 70 wt.% revealed the presence of several small, isolated areas of damage. This damage pattern was present on at least one TP of each panel but was more pronounced and noticeable on the TPC panels at loading of 70 wt.%. In the aviation industry, when the lightning strike takes place, the lightning current is expected to be on the top surface of the fiber composites; thus, electrically and thermally conductive nanocomposite hard coatings applied on the composite top surfaces in this study will minimize the electrical, thermal and other structural and surface damages on the composites.

**Table 6:** Comparison of fiber damage between Ag20/Cu at loading of 70 wt.% panels and Ag30/Cu at loading of 70 wt.% panels

Panel ID	Ag/Cu	Wt%	Dielectric Layer	Test Pt	Delamination: Front			Delamination: Back		
					Longitudinal (in)	Transverse (in)	Area (in^2^)	Longitudinal (in)	Transverse (in)	Area (in^2^)
2	20	55		1	3.25	2.5	8.125	11.5	1.5	17.25
2	20	55		1	1	1	**1**	0	0	0
2	20	55		2	3.25	4	13	1	2.75	2.75
2	20	55		***2***	0	0	0	0	0	0
3	20	70		1	3.75	5	18.75	0	0	0
3	20	70		2	3	3.2	9.6	1	0	0
5	30	55		1	4	7–75	11	0	0	0
5	30	55		1	0	0	0	0	0	0
5	30	55		2	3.75	3	11.25	0.25	0.5	0.125
6	30	70		1	4.25	3.25	13.8125	0	0	0
6	30	70		2	3	3.5	10.5	^0^	^0^	^0^

**Table 7:** Comparison of fiber damage between Ag20/Cu at loading of 70 wt.% panels and Ag30/Cu at loading of 70 wt.% panels

Panel ID	Ag/Cu	Wt%	Dielectric Layer	Test Pt	Fiber Breakage: Front			Fiber Breakage: Back		
					Longitudinal (in)	Transverse (in)	Area (in^2^)	Longitudinal (in)	Transverse (in)	Area (in^2^)
2	20	55		1	3	2.25	6.75	0.25	0.25	0.0625
2	20	55		1	0.125	0.5	0.0625	0	0	0
2	20	55		2	2.5	2.25	5.625	0	0	0
2	20	55		2	0.25	0.25	0.0625	0	0	0
3	20	70		1	2.75	3	8.25	0	0	0
3	20	70		2	2.75	3.5	9.625	0	0	0
5	30	55		1	3	2.5	7.5	0	0	0
5	30	55		1	0	0	0	0	0	0
5	30	55		2	2.25	3	6.75	0	0	0
6	30	70		1	4.25	3.25	13.8125	0	0	0
6	30	70		2	3	3.5	10.5	0	0	0

**Fig. 14:**
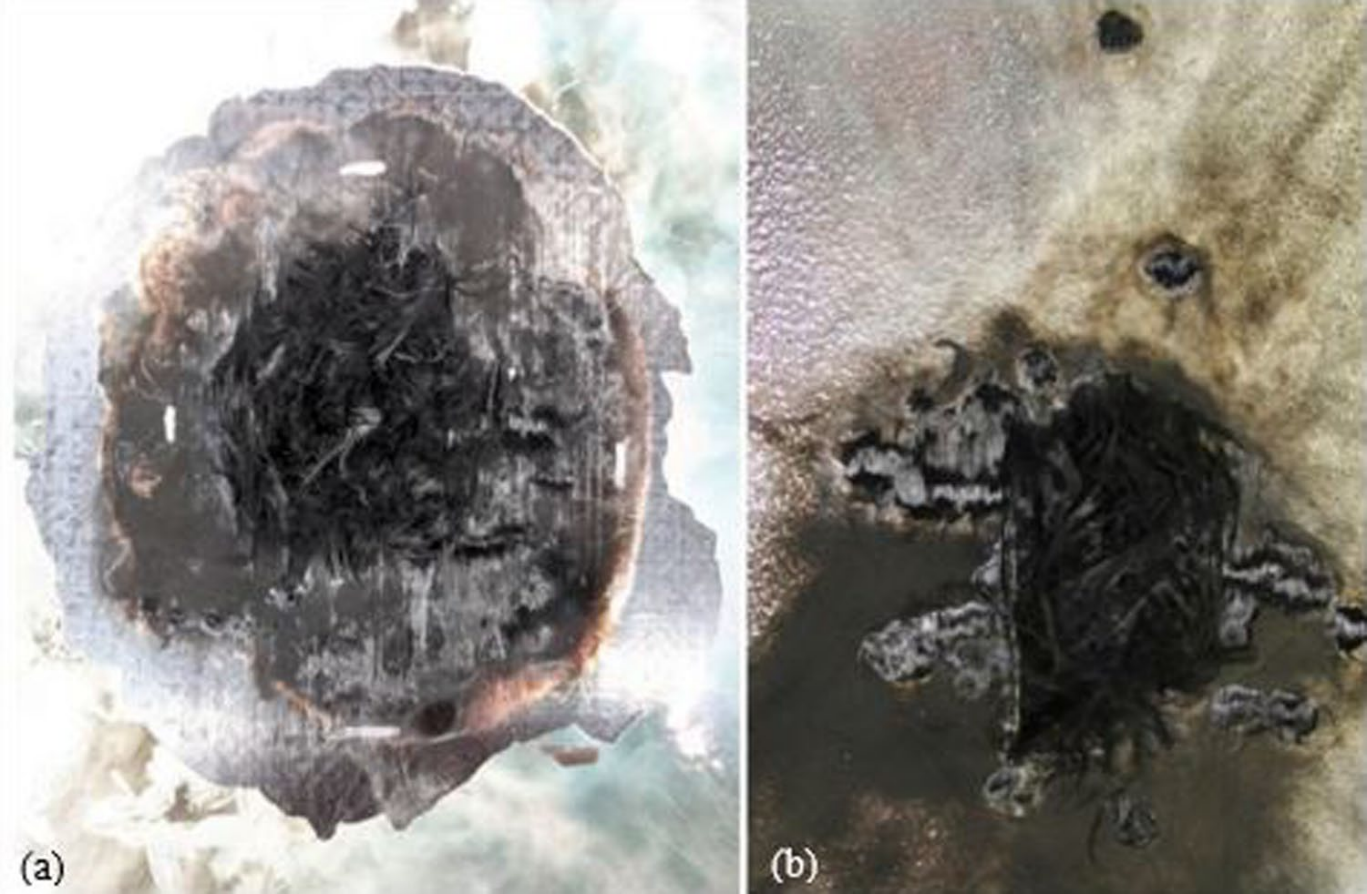
Outside damage morphology images after lightning strike test **a** Panel 5 (Ag30/Cu at loading of 55 wt.%); and Panel 6 (Ag30/Cu at loading of 70 wt.%)

Figure 14 illustrates the contrast in the exterior damage morphology between Panel 6 (Ag30/Cu at loading of 70 wt.%) in Fig. 14a and Panel 5 (Ag30/Cu at loading of 55 wt.%) in Fig. 14b. It is crucial to recall Fig. 7. The cross-section analysis of the 70 wt.% loaded panels revealed significant variations in the thickness of the nanofiller, which may have impacted the results and contributed to the observed differences in damage morphology. It is plausible that the proximity of the conductive nanofiller to the paint surface facilitated the formation of multiple leaders that could disperse the arc root. The micrographs support this idea from Fig. 7, which illustrates that the conductive nanofiller layer in the TPC panels at loading of 70 wt.% was situated closer to the surface than the layer in the TPC panels at loading of 55 wt.%. As a result, multiple leaders could form and reach upwards, distributing the current density over a wider area of the panel. This phenomenon would result in dividing the lightning strike energy into multiple focal points, which explains that TPC panels at loading of 70 wt.% consistently perform better despite having nearly 40% less conductive material.

#### Comparisons of HBN dielectric to non-dielectric situations

The comparison between TPC panels containing an HBN dielectric sublayer and those without did not show a clear trend in materials responses for this work. The only distinguishing feature between the two types of panels was the absence of a hole in either of the HBN dielectric panels. However, inside delamination was observed in both Ag/Cu20 and Ag/Cu30 panels. Due to the limited amount of information available, it is impossible to make a definite statement about the effectiveness of HBN dielectric as a layer of protection against lightning strikes. However, it is assumed that the HBN dielectric layer may provide some level of insulation and impedance to current flow under laboratory conditions, its efficacy in withstanding the high voltages and energy levels associated with natural lightning strikes is limited. During a natural lightning strike, the voltage levels can reach several million volts, far exceeding the capabilities of typical laboratory equipment. As a result, the driving voltage of the HBN dielectric, which is likely to be surpassed. This can lead to the formation of electrical arcs and the penetration of the dielectric layer, allowing lightning current to pass through and potentially causing damage to underlying structures. While HBN dielectric layers may offer some degree of protection against low-voltage electrical discharges and transient events, they are generally not sufficient to withstand the extreme conditions associated with lightning strikes. Additionally, since there is no clear or less evidence of improved performance, the increased weight of the LSP due to adding the HBN dielectric is a significant disadvantage. This is particularly important in the commercial aircraft industry, where the cost of adding weight is very high for fuel consumption.

Our experimental investigations demonstrate that the incorporation of conductive nanofillers yields a substantial reduction in lightning strike damage observed in spray-coated TPC panels. This notable mitigation can be primarily attributed to the enhancement in electrical conductivity facilitated by the presence of these nanofillers. Despite the significant reductions in lightning strike damage observed in TPCs through the incorporation of conductive nanofillers, achieving complete elimination of such damage remains a formidable challenge. This challenge persists due to the insufficiency of the improvement in electrical conductivity attained solely using nanofillers. It is imperative to note that the specimens utilized in this study were not subjected to electrical characterization tests, primarily due to the constrained scope of the project. However, it is worth mentioning that extensive experimental testing data are readily available in the existing literature. The intuitive approach to enhancing electrical conductivity in TPCs involves increasing the weight fraction of conductive nanofillers. Nonetheless, this strategy presents a dilemma as it entails alterations in numerous other properties of the epoxy resin mixture. While increasing the weight fraction of nanofillers may indeed lead to improved electrical conductivity, it also introduces changes in a multitude of material characteristics. These changes encompass several aspects, notably the elevation of viscosity, alterations in rheological properties such as storage and loss modulus, and the prolongation of gel time. Elevated viscosity levels pose practical challenges, rendering the epoxy resin mixture less conducive to the manufacturing process of TPCs, particularly when employing resin infusion techniques. Furthermore, observations have highlighted that those modifications in the rheological behavior of the epoxy system, coupled with the decreased gel time, have been implicated in the emergence of defects within the material structure. These defects encompass a range of imperfections, including voids, bubbles, and various structural discontinuities. Such anomalies can exert detrimental effects on both the thermal and electrical conductivities of the TPC.

Furthermore, in the application of conductive nanofillers for LSP, there exists a significant challenge in striking a balance between performance optimization and cost considerations. This trade-off represents a formidable barrier impeding the widespread adoption of this technology in practical applications. This situation arises primarily from the significant expense associated with materials, exceptionally pure silver (Ag), as well as the constraints in achieving substantial performance improvements in LSP. Despite efforts, the technology has yet to eradicate lightning strike damage. Future research endeavors should delve into methodologies aimed at increasing the weight fractions of nanofillers within TPCs. Concurrently, there is a need for modeling studies to comprehensively elucidate the impact of nanofiller weight fractions on the overall anisotropic electrical conductivity of the TPC material. At present, the incorporation of nanofillers into TPCs is constrained by several factors, including alterations in rheological properties upon admixture with epoxy resin and the possibility of nanofiller agglomeration. These limitations impose restrictions on the achievable weight fractions of nanofillers within the composite matrix. Furthermore, there is a pressing need to develop computational models that can facilitate the determination of optimal nanofiller weight fractions aimed at mitigating lightning strike damage. In conclusion, it is strongly suggested that future researchers delve into examining the influence of nanofillers on the microstructure and mechanical properties of TPC materials. These investigations should encompass an analysis of parameters such as porosity, defects, translaminar fracture toughness, and delamination resistance. The goal is to ascertain whether the incorporation of nanofillers maintains, or preferably enhances, the mechanical integrity of the composite without compromising its structural properties.

## Conclusions

The conductive nano and sub-micro fillers were applied on the thermoplastic composite surfaces by an epoxy resin-based matrix, Ag20/Cu and Ag30/Cu, spray coating process. Two nanofillers that had low resistance after being cured in the epoxy matrix were chosen to be spray coated on TPC panels at loadings of 55 wt.% and 70 wt.%. Three TPC panels were coated with Ag20/Cu and three with Ag30/Cu. The first panel of each set was treated with an HBN dielectric at loading of 20 wt.%, followed by the coating with Ag/Cu (Ag20/Cu and Ag30/Cu) at loading of 55 wt.%. The second panel of each set was coated solely with Ag/Cu at loading of 55 wt.%, while the third panel was coated with Ag/Cu at loading of 70 wt.%. The process of spray coating the Ag/Cu at loading of 70 wt.% panels proved challenging, resulting in conductive coatings that were poorly packed and thinly spread compared to the Ag/Cu at loading of 55 wt.% panels. Although the spray coating process for the Ag/Cu at loading of 70 wt.% panels resulted in a loosely packed and thin conductive coating compared to the Ag/Cu at loading of 55 wt.% panels, they were still able to resist the interior damage and delamination. This could be because the loosely packed coating of the Ag/Cu at loading of 70 wt.% panels was closer to the paint surface, which enabled greater dispersal of the arc root and an even distribution of the energy across the entire panel surface. In the TPC panels, lightning damage manifested primarily in the form of fiber damage and some delamination, with varying degrees of severity assigned to each type of damage. Specifically, the lightning attachment point exhibited a significant degree of fiber damage and delamination that spanned multiple layers, with the damaged area extending all the way to the panel's edge. Additionally, several piles of flaky black carbon powder and residual melted material were observed to have accumulated on the panel surface as a result of the lightning strikes. The formation of material piles on the TPC panel surface was attributed to the sublimation of fibers and the pyrolytic carbonation process that occurred due to the extreme temperatures generated by the lightning arc. In terms of the extent of damages caused to the TPC panels, it was observed that lightning current components D and B had a particularly significant impact, along with component C* to a lesser degree, with the depth of damage varying based on the specific combination of components present during the lightning strike. One particular test was performed on Panel 2, which was coated with Ag20/Cu at loading of 55 wt.% panels, resulting in a hole through the panel. Incorporating a dielectric in the form of HBN did not positively or negatively impact the effectiveness of the LSP system. Therefore, the use of the dielectric was determined to be an additional unnecessary weight without any beneficial effects. With further refinement, the spray-coated LSP using nanoflakes of Ag/Cu could potentially serve as a cost-effective alternative to the traditional LSP for TPC aircraft components.

## Data Availability

All data underlying the results are available as part of the article and no additional data source are required.
